# Electron transport chain: a crucial target of luteolin in combating *Trueperella pyogenes*

**DOI:** 10.1128/spectrum.01985-26

**Published:** 2026-10-15

**Authors:** Yuru Guo, Yueting Guo, Ruxia Wang, Simiao Wang, Hanyu Zhao, Yingyu Wang, Shengli Niu, Chunlian Tian, Mingchun Liu

**Affiliations:** 1 Key Laboratory of Livestock Infectious Diseases, Ministry of Education, College of Animal Science and Veterinary Medicine, Shenyang Agricultural University 98428, Shenyang, China; 2 Key Laboratory of Ruminant Infectious Disease Prevention and Control (East), Ministry of Agriculture and Rural Affairs, College of Animal Science and Veterinary Medicine, Shenyang Agricultural University 98428, Shenyang, China; UJF-Grenoble 1, CHU Grenoble, Grenoble, France

**Keywords:** *Trueperella pyogenes*, luteolin, electron transport chain, oxidative phosphorylation, energy metabolism

## Abstract

*Trueperella pyogenes* is an opportunistic pathogen capable of causing infections in both domestic and wild animals. Furthermore, the dissemination of resistance genes has resulted in varying degrees of antimicrobial resistance among *T. pyogenes* isolates. Therefore, it is necessary to develop new anti-*T*. *pyogenes* drugs to address its increasing resistance. Luteolin, a natural flavonoid, has been found to exhibit good antibacterial activity against *T. pyogenes* in our previous research. This study aims to utilize high-throughput proteomics technology to identify potential novel targets and illuminate the action mechanism of luteolin. Our results indicated that luteolin could target electron transport chain complexes I, II, and V to inhibit the oxidative phosphorylation of *T. pyogenes*. Besides, luteolin could inhibit the enzyme activities involved in glycolysis and the tricarboxylic acid cycle, which eventually leads to a decrease in reduced hydrogen supplied to the electron transport chain and reduces the content of intracellular adenosine triphosphate. Based on the high-throughput proteomic analysis and multiple experimental results, the inhibition of electron transport chain complexes appears to be a significant mechanism by which luteolin exerts its antibacterial effect against *T. pyogenes*. Overall, this study provides a new insight into the anti-*T*. *pyogenes* mechanism of luteolin and offers new targets to develop anti-*T*. *pyogenes* drugs.

**IMPORTANCE:**

*Trueperella pyogenes* is an opportunistic pathogen that can cause suppurative infections in multiple animals. Its existing resistance may bring challenges to anti-infection therapy. As a promising natural antibacterial agent, luteolin shows potent activity against this bacterium. This study revealed that luteolin disrupts bacterial energy metabolism by targeting electron transport chain complexes, thereby reducing intracellular adenosine triphosphate content. Our findings expanded the new antibacterial mechanism of luteolin and identified novel targets. This work provides valuable references for developing novel drugs against drug-resistant bacterial infections by targeting the electron transport chain.

## INTRODUCTION

*Trueperella pyogenes*, formerly classified as *Corynebacterium pyogenes*, is a gram-positive, short rod-shaped, non-spore-forming, and non-motile bacterium that can grow under aerobic and anaerobic conditions (1, 2). *T. pyogenes* is a commensal and opportunistic pathogen. This bacterium can cause suppurative infections such as mastitis, endocarditis, pneumonia, arthritis, and liver abscess in numerous domestic animals and wild animals, including swine, cattle, goats, sheep, and forest musk deer. Diseases caused by *T. pyogenes* lead to significant economic losses, especially in dairy cows and swine breeding, causing a reduction in milk production and meat quality (1–4). Cases of *T. pyogenes* infection in humans are quite rare and mostly limited to sporadic case reports, including conditions such as respiratory infections, skin ulcer, endocarditis, septicemia, arthritis, and pneumonia. The latest literature also reported the first case of *T. pyogenes* infection in the human head and neck. Human infections caused by *T. pyogenes* are often related to contact with animals and mainly in patients with an impaired immune system (5–7).

At present, the treatment of diseases caused by *T. pyogenes* mainly relies on traditional antimicrobial drugs, such as β-lactams, tetracyclines, macrolides, and fluoroquinolones (8). However, extensive use of antibiotics has led to the development of bacterial resistance. *T. pyogenes* strains with varying degrees of resistance to antibiotics, including multidrug-resistant strains, have been isolated worldwide, which poses a great challenge to the treatment of infectious diseases caused by *T. pyogenes* (1, 2, 9, 10). Previous reports have indicated that numerous resistance genes conferring resistance to macrolides, tetracyclines, aminoglycosides, and sulfonamides, such as *ermB*, *tetM*, *tetW*, *aacC*, *aadA*, *folP*, and *sul1*, are widely detected in *T. pyogenes* isolates (11–13). The prevalence of these resistance genes poses potential risks to the treatment of *T. pyogenes* infections using traditional antimicrobial agents. Therefore, it is necessary to develop novel antimicrobial agents to combat *T. pyogenes* infections.

Antimicrobial compounds from plants could serve as substitutes for antibiotics in fighting drug-resistant bacterial infections (14). Luteolin is a natural flavone existing in various plants such as *Apium graveolens*, *Thymus vulgaris*, *Chrysanthemum morifolium*, *Lonicera japonica*, and *Laurus nobilis*, among others. It has been reported that luteolin is abundant in *T. vulgaris* (36 mg/100 g DW) and *L. nobilis* (393.4 mg/100 g DW). Luteolin has multiple pharmacological activities, including antibacterial, anti-inflammatory, antioxidant, and antitumor properties (15). Studies have shown that luteolin exhibits antibacterial activity against a variety of gram-positive and gram-negative bacteria, such as *Listeria monocytogenes*, *Staphylococcus aureus*, *Escherichia coli*, and *Salmonella typhimurium* (16–18). Although previous works did not find that luteolin was the best antibacterial compound among flavonoids (19, 20), our previous work showed that among the tested compounds, luteolin, apigenin, baicalin, nobiletin, and quercetin against *T. pyogenes*, luteolin was the most active one against this bacterium.

Luteolin is a widely distributed natural flavonoid with favorable safety profiles, but it exhibits limited oral bioavailability due to poor water solubility and rapid intestinal metabolism, which consequently affects its biological properties and efficacy. Several drug delivery strategies, such as liposomes, have been developed to raise its bioavailability (21). Current published data on its tissue distribution are insufficient to confirm whether it can achieve effective concentrations at the primary anatomical sites of animal *T. pyogenes* infection. Nevertheless, our previous studies have demonstrated that luteolin can effectively treat *T. pyogenes* infection in a rat model (22). Further *in vivo* tests and optimized delivery formulations will be necessary to overcome pharmacokinetic limitations before clinical application.

Proteomics is a high-sensitivity and high-throughput approach that has been widely applied for the identification of drug targets and exploring the mechanisms of action of different drugs. Given that proteins serve as pivotal functional executors in biological systems, this study employed high-throughput tandem mass tag (TMT)-based quantitative proteomics to systematically analyze the effects of luteolin on the proteome of *T. pyogenes*. By integrating a global profiling approach with experimental biochemical and molecular biological assays, we aimed to identify potential novel targets of luteolin in combating *T. pyogenes*, demonstrating its potential application in *T. pyogenes* infection treatment.

## MATERIALS AND METHODS

### Source of luteolin and test strain

**Fig 1:**
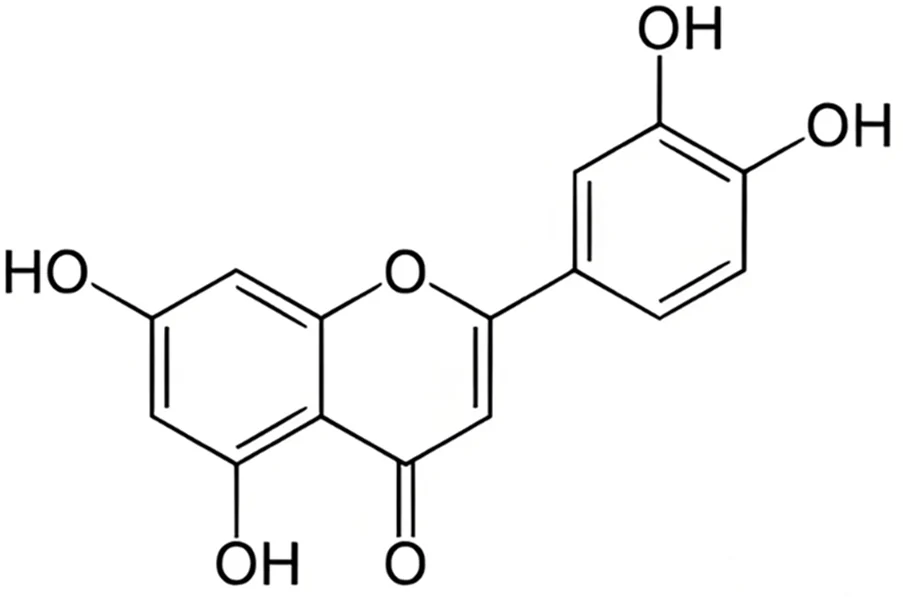
The chemical structure of luteolin.

Luteolin (98% purity) was purchased from Shanghai PureOne Biotechnology Co., Ltd. (Shanghai, China). Its chemical structure is shown in Fig. 1. The resistant *T. pyogenes* strain BM-H06-3 was isolated from dairy cows with endometritis and stored in our laboratory. The antimicrobial susceptibility profile of *T. pyogenes* BM-H06-3 is shown in Table S1. The culture method for *T. pyogenes* was performed as previously described (23).

### Cultivation and pre-treatment of *T. pyogenes*

The *T. pyogenes* BM-H06-3 was cultured to the logarithmic growth phase and diluted in 100 mL NB medium (1:100, vol/vol) to obtain the bacterial suspensions (approximately 1 × 10^6^ CFU/mL). Then the bacterial suspensions were treated with luteolin at a final concentration of 1/2 MIC (78 μg/mL) (23) and further cultured at 37°C for 36 h. *T. pyogenes* suspensions without luteolin under the same culture conditions were used as control. Each group was prepared in three technical replicates. For convenience, the luteolin-treated groups were defined as treatment-1, treatment-2, and treatment-3, while the control groups were defined as control-1, control-2, and control-3.

### TMT labeling and LC-MS/MS analysis

The bacterial cells were harvested and lysed via intermittent sonication in SDT lysis buffer. After the lysate was centrifuged at 14,000 × *g* for 40 min, the supernatant containing total proteins was filtered and quantified by the BCA method. The protein samples were digested by trypsin using the filter-aided sample preparation method and labeled using TMT reagent following the manufacturer’s guidelines (Thermo Fisher Scientific, Waltham, MA, USA). The TMT-labeled digest samples were mixed at equal amounts and then fractionated into 10 fractions. Each fraction underwent Nano LC-MS/MS analysis. LC-separated samples were analyzed on a Q Exactive mass spectrometer (Thermo Fisher Scientific). The MS/MS data were analyzed using the MASCOT engine (v2.2). Tandem mass spectra were searched against the UniProt database of *Trueperella pyogenes* (5,405 entries). The screening criteria for credible peptides was false discovery rates ≤0.01. The protein ratios were calculated according to the only unique peptides. The proteins with a 1.2-fold change and a *P* value of <0.05 were considered as differentially expressed proteins (DEPs).

### Bioinformatics analysis

Cluster 3.0 and the Java TreeView software were used to perform hierarchical clustering analysis. Blast2Go (v3.3.5) was used for Gene Ontology (GO) mapping and annotation of identified proteins. The Kyoto Encyclopedia of Genes and Genomes (KEGG) database (http://www.genome.jp/kegg/) was used for metabolic pathway annotation of identified proteins. GO and KEGG pathway enrichment analyses of DEPs were applied based on Fisher’s exact test.

### Quantitative real-time PCR

Several DEPs related to oxidative phosphorylation, tricarboxylic acid (TCA) cycle, and glycolysis obtained by TMT methods were further analyzed at the mRNA level by quantitative real-time PCR (qRT-PCR). The details of qRT-PCR primers are listed in Table S2. The protocol for luteolin treatment of *T. pyogenes* was described previously. The total RNA of *T. pyogenes* was extracted using TRIzol Reagent (Ambion, Carlsbad, CA, USA). The transcription levels of selected genes were measured on the QuantStudio 3 Real-Time PCR System (Applied Biosystems, Foster, CA, USA). The mRNA expression patterns of selected genes were normalized against the *gapA* gene and quantified using the 2^−ΔΔCt^ method.

### Measurement of the energy metabolism level

The oxygen consumption rate (OCR) and the extracellular acidification rate (ECAR) were measured to evaluate the energy metabolism level of *T. pyogenes* on a Seahorse XFe96 Extracellular Flux Analyzer (Agilent Technologies, Santa Clara, CA, USA) as previously described with minor modifications (24). After co-incubation of *T. pyogenes* with luteolin at 37°C at different times (12, 24, and 36 h), bacterial cells were collected and resuspended with fresh NB medium to achieve an OD_600_ of 0.15. Subsequently, 100 μL of bacterial suspension was added to the cell culture microplates pre-coated with Cell-Tak (Corning, Corning, NY, USA). The plates were centrifuged at 1,400× *g* for 10 min to facilitate cell attachment and generate a monolayer from the suspension. Subsequently, 80 μL of NB medium was added to bring up to a final volume of 180 μL. The OCR and ECAR were monitored over time in the flux analyzer.

### Determination of intracellular adenosine triphosphate contents

After *T. pyogenes* was incubated with luteolin at different times (12, 24, and 36 h), the bacterial pellets were harvested and lysed via intermittent sonication (power 20%, 2 s on and 1 s off, total of 1 min) in 1 mL of lysis buffer. The supernatant was collected by centrifugation at 4°C, 10,000 × *g* for 10 min. The content of intracellular adenosine triphosphate (ATP) was determined using an ATP Assay Kit (Solarbio, Beijing, China) according to the manufacturer’s protocols.

### Determination of electron transport chain complex I–V activities

After *T. pyogenes* was incubated with luteolin at different times (12, 24, and 36 h), the activities of electron transport chain complex I (NADH dehydrogenase), complex II (succinate dehydrogenase [SDH]), complex III (cytochrome C reductase), complex IV (cytochrome C oxidase), and complex V (ATP synthase) were determined using the Electron Transport Chain Complex I–V Activity Assay Kit (Solarbio), following the manufacturer’s protocols (25).

### Molecular docking of luteolin and electron transport chain complex subunits

Molecular docking was performed to investigate the potential interactions between luteolin and the electron transport chain complex subunits whose expression was significantly down-regulated after luteolin treatment. The 3D structures of these subunits were generated through homology modeling using the SWISS-MODEL server (https://swissmodel.expasy.org/) based on their respective amino acid sequences (Table S3), with resulting models saved in PDB format. The 3D structure of the protein was imported into PyMOL 2.3.0 to remove crystallographic water molecules and native ligands. Subsequently, the processed protein structure was loaded into AutoDock Tools (v1.5.6) for hydrogen addition, charge calculation, charge assignment, and atom-type specification, followed by saving the file in pdbqt format. The 3D structure of luteolin was downloaded from the PubChem database (CID: 5,280,445) and imported into ChemBio3D Ultra 14.0 for energy minimization. Molecular docking was performed using AutoDock Vina 1.1.2 to evaluate the interaction between luteolin and transport chain complex subunits. During molecular docking, the exhaustiveness for global search was set to 32, and the remaining parameters were kept at their default values. The optimal binding conformation was selected based on the predicted binding energy. The results of molecular docking were visualized and analyzed for interaction patterns using PyMOL 2.3.0 and Ligplot 2.2.5.

### Molecular dynamics simulations

Molecular dynamics (MD) simulations of NuoF-luteolin and AtpA-luteolin complexes were conducted using GROMACS (v2022.3). NADH-ubiquinone oxidoreductase subunit F (electron transport chain complex I subunit, abbreviated as NuoF) and ATP synthase subunit α (electron transport chain complex V subunit, abbreviated as AtpA), which displayed superior binding energy with luteolin in molecular docking, were chosen for MD simulation following the previously described method (26). The trajectories obtained from the completed 100 ns MD simulation were utilized for analyzing key structural parameters.

### Prokaryotic expression and purification of electron transport chain complex subunits

Based on the results of molecular docking and MD simulations, NuoF and AtpA were expressed and purified for subsequent affinity tests with luteolin. Briefly, the PCR products of *nuoF* and *atpA* were ligated with pET-28a (+) vector to construct the recombinant plasmids pET-28a-*nuoF* and pET-28a-*atpA*. The BL21 (DE3) *E. coli* cells transformed with recombinant plasmids were induced to express the fusion proteins His-NuoF and His-AtpA using lactose at a final concentration of 10 g/L. The soluble fusion proteins were purified using His GraviTrap (Cytiva, Uppsala, Sweden) based on the principle of immobilized metal ion affinity chromatography. The recombinant proteins obtained were characterized by Western blot assay.

### Affinity detection of luteolin and electron transport chain complex subunits

The interaction of luteolin with His-NuoF and His-AtpA, respectively, was detected using the Biacore T200 system (Cytiva) based on the principle of surface plasmon resonance (SPR). The His-NuoF and His-AtpA fusion proteins were diluted into a solution at a concentration of 50 μg/mL using 1× PBS-P buffer, and luteolin was prepared at a concentration of 200 μM using 1× PBS-P buffer and diluted in a gradient to obtain luteolin at a concentration of 1.56–50 µM. The ligands His-NuoF and His-AtpA were coupled to the surface of the Series S Sensor Chip NTA (Cytiva). After that, the analyte luteolin was flowed over the chip surface in descending order. After completion of the test, Biacore T200 Evaluation Software 3.2.1 was used for kinetic/affinity fitting.

### Measurement of enzyme activities related to glycolysis and TCA cycle

After co-incubation of *T. pyogenes* with luteolin at 37°C for 12, 24, and 36 h, respectively, enzymatic activities related to energy metabolism were measured using pyruvate kinase (PK), fructose phosphate kinase (PFK), citrate synthase (CS), and SDH activity detection kits (Solarbio) according to the manufacturer’s instructions.

### Statistical analysis

Each experiment was performed in triplicate. Data were presented as the mean ± standard deviation. Software SPSS 20.0 was used to perform Student’s *t*-test. Asterisks indicate statistically significant differences between groups (*0.01 < *P* < 0.05 and **P < 0.01).

## RESULTS

### Identification and analysis of DEPs after luteolin treatment

**Fig 2:**
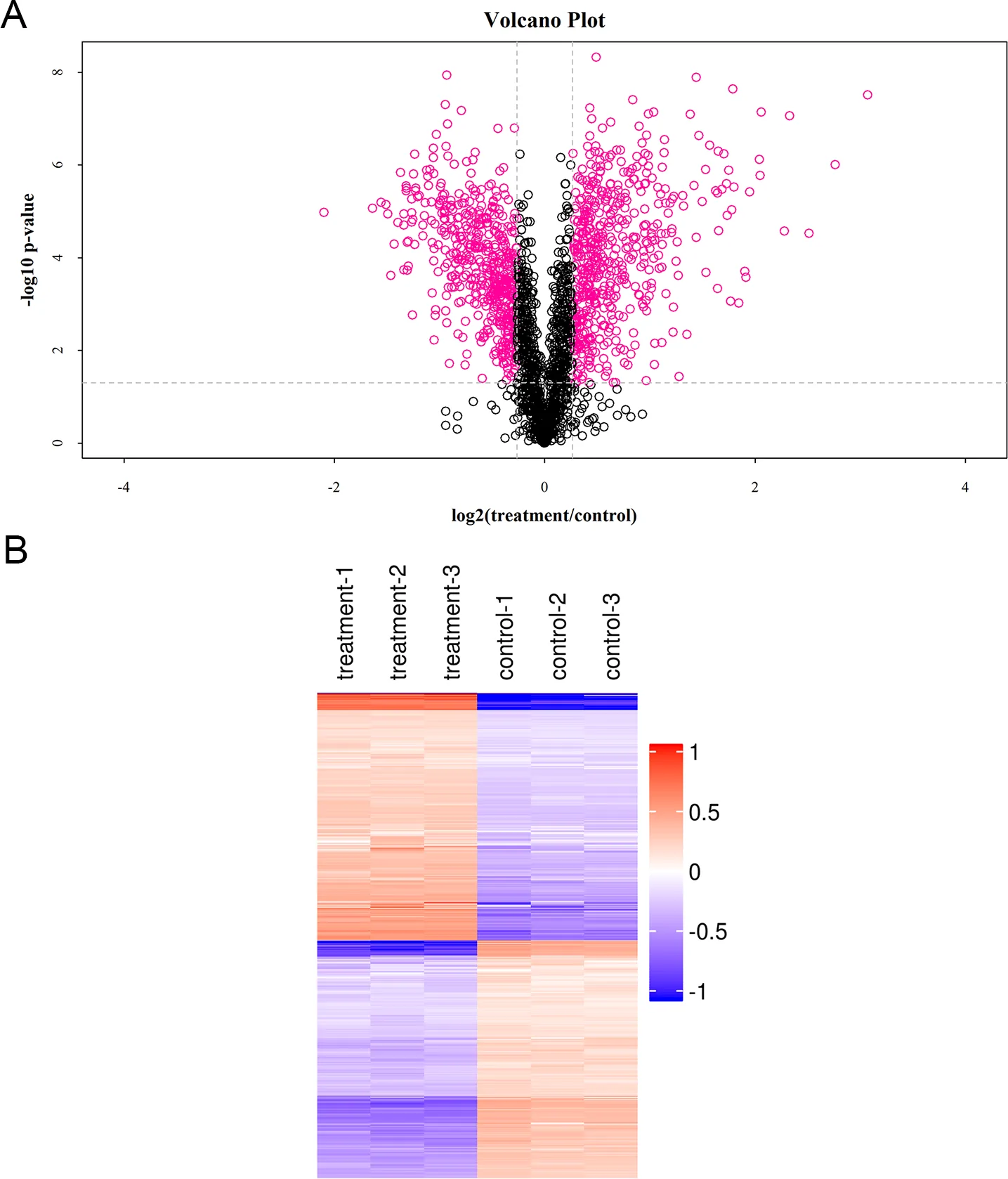
TMT-based proteomic analysis reveals DEPs between the luteolin treatment group and the control group. (**A**) Volcano plot of DEPs. (**B**) Cluster analysis results of DEPs.

TMT-based quantitative proteomics was applied to analyze DEPs between the luteolin treatment and control groups. A total of 2,057 *T. pyogenes* proteins were identified in this study. As shown in the volcano plot of DEPs, there were a total of 1,054 DEPs, among which 536 were upregulated and 518 were downregulated (Fig. 2A). The heat map of hierarchical clustering of DEPs revealed that the expression patterns of luteolin-treated *T. pyogenes* samples were significantly altered, and there were both upregulated and downregulated proteins (Fig. 2B). Details for partial DEPs are provided in Table S4.

### GO analysis of DEPs

**Fig 3:**
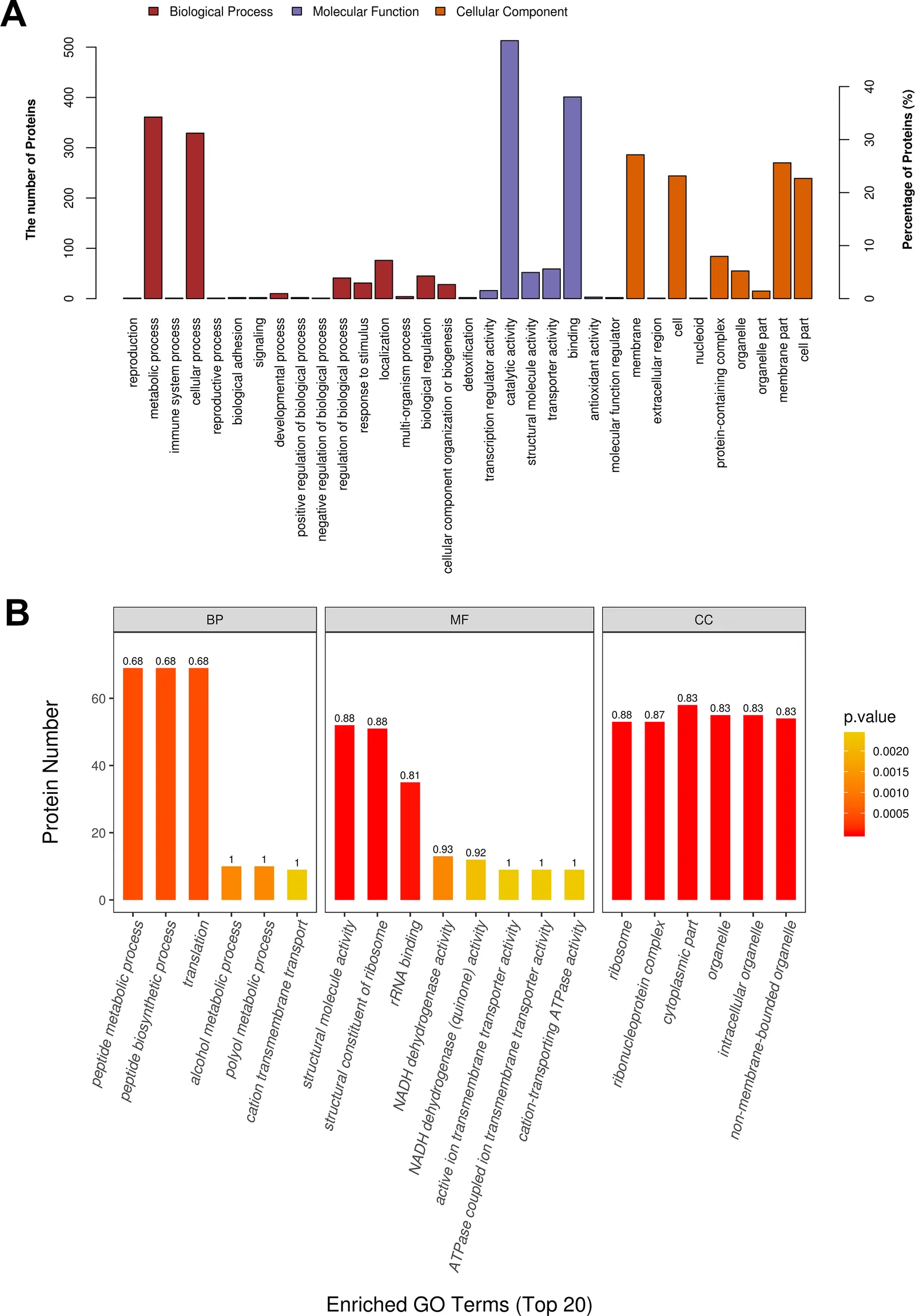
GO annotation (**A**) and enrichment analysis (**B**) of DEPs between the luteolin treatment group and the control group.

The DEPs were annotated into three GO categories, including molecular function (MF), biological process (BP), and cellular component (CC) (Fig. 3A). In BP, the DEPs were associated with 17 terms, primarily metabolic process (38.5%) and cellular process (35.1%). For CC, the DEPs mapped to nine terms, with the highest representation in membrane (23.9%), membrane part (22.6%), cell (20.4%), and cell part (20.0%). In MF, catalytic activity (49.0%) and binding (38.3%) were the most abundant terms among the seven identified.

GO enrichment analysis revealed 84 significantly enriched terms (Fisher’s exact test), with the top 20 displayed in Fig. 3B. The DEPs were significantly associated with molecular functions such as structural constituent of ribosome, rRNA binding, NADH dehydrogenase activity, NADH dehydrogenase (quinone) activity, ATPase-coupled ion transmembrane transporter activity, and cation-transporting ATPase activity. These findings suggested that luteolin may interfere with ribosomal function and energy metabolism in *T. pyogenes*.

### KEGG pathway analysis of DEPs

**Fig 4:**
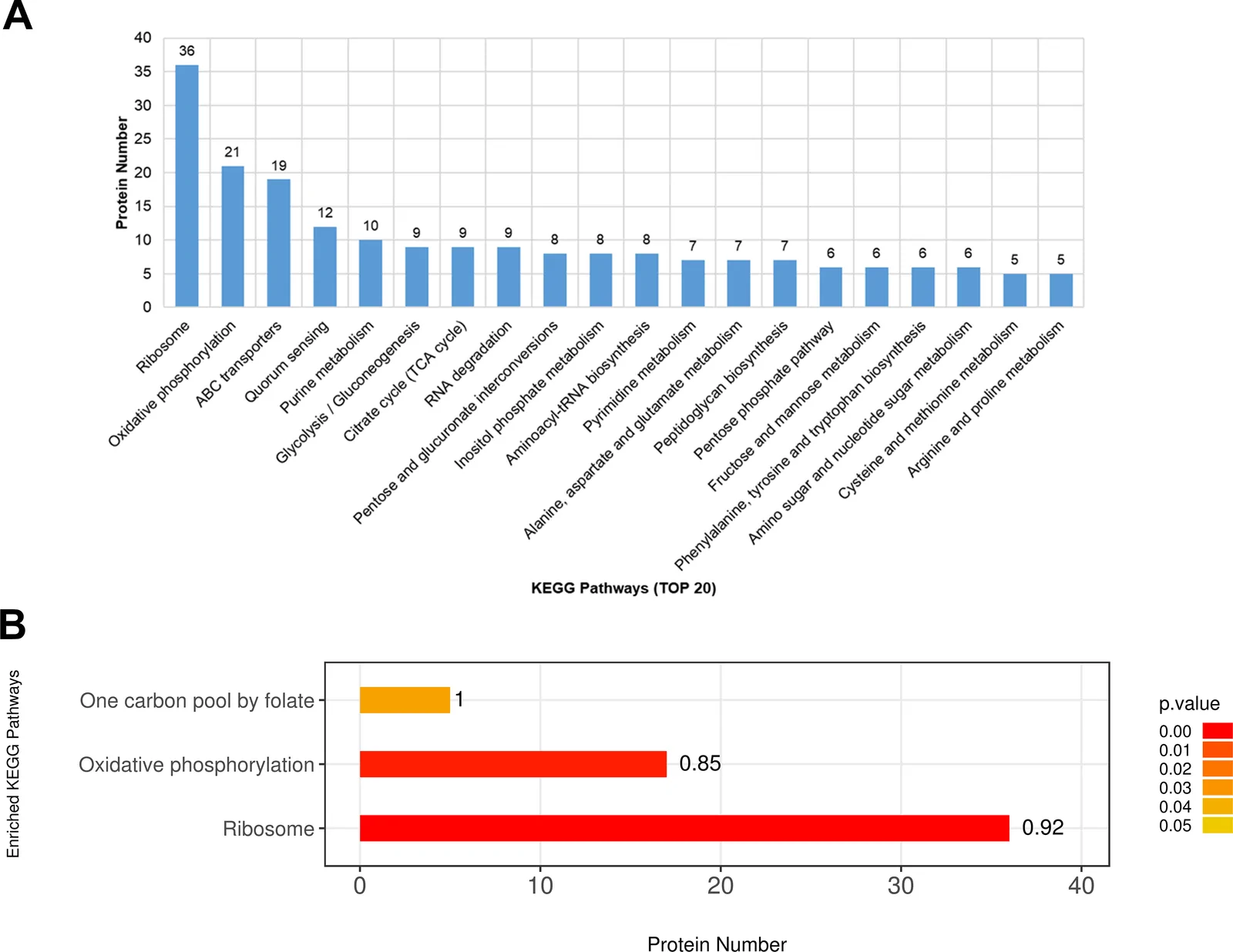
KEGG pathway annotation and enrichment analysis of DEPs between the luteolin treatment group and the control group. (**A**) The number of DEPs contained in different KEGG metabolic pathways. (**B**) The significance level of KEGG pathway enrichment. The color gradient represents the size of the *P* value. The color changes from orange to red, and the closer to red, the smaller the *P* value.

KEGG pathway analysis revealed that the DEPs were associated with 107 metabolic pathways, and the top 20 pathways with the largest number of DEPs are presented in Fig. 4A. The ribosome pathway contained the highest number of DEPs, followed by oxidative phosphorylation and ABC transporter pathway.

**Fig 5:**
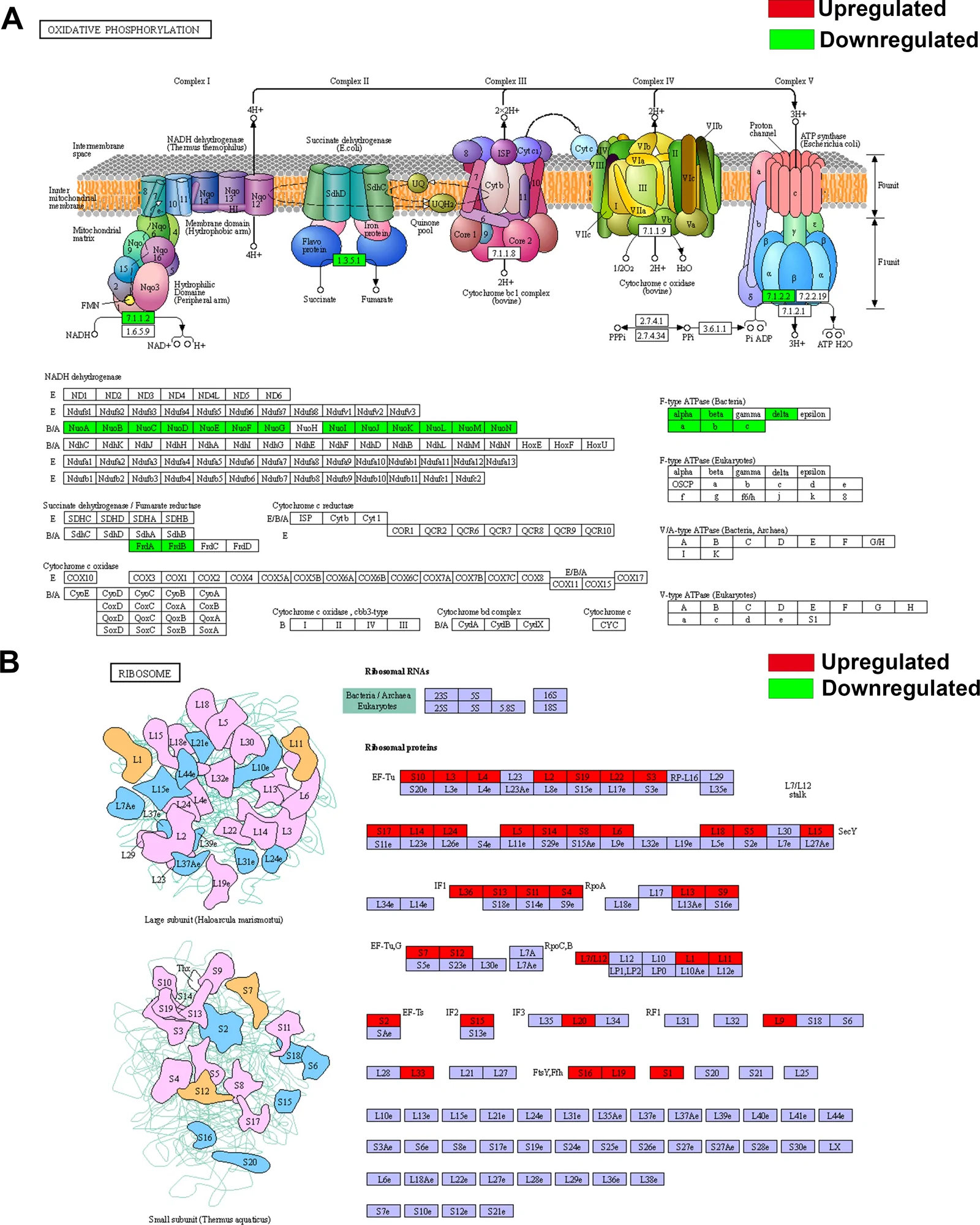
DEPs in the oxidative phosphorylation pathway and the ribosome pathway (downloaded from the KEGG database, green frames represent downregulated proteins, and red frames represent upregulated proteins). (**A**) The DEPs enriched in the oxidative phosphorylation pathway were downregulated. (**B**) The DEPs enriched in the ribosome pathway were upregulated.

KEGG enrichment analysis identified three significantly affected pathways (Fig. 4B), with the ribosome pathway exhibiting the lowest *P*value, followed by oxidative phosphorylation and one-carbon pool by folate. Consistent with the annotation results, the ribosome and oxidative phosphorylation pathways contained the highest numbers of DEPs, indicating that luteolin strongly impacted ribosomal function and energy metabolism in *T. pyogenes*. Strikingly, all DEPs in the oxidative phosphorylation pathway were downregulated, including the subunits of electron transfer chain complexes I, II, and V (Fig. 5A), while those in the ribosome pathway were upregulated (Fig. 5B). Given that oxidative phosphorylation is essential for ATP production, our findings demonstrated that the disruption of electron transfer chain complexes by luteolin may greatly contribute to its anti-*T*. *pyogenes* activity.

**Fig 6:**
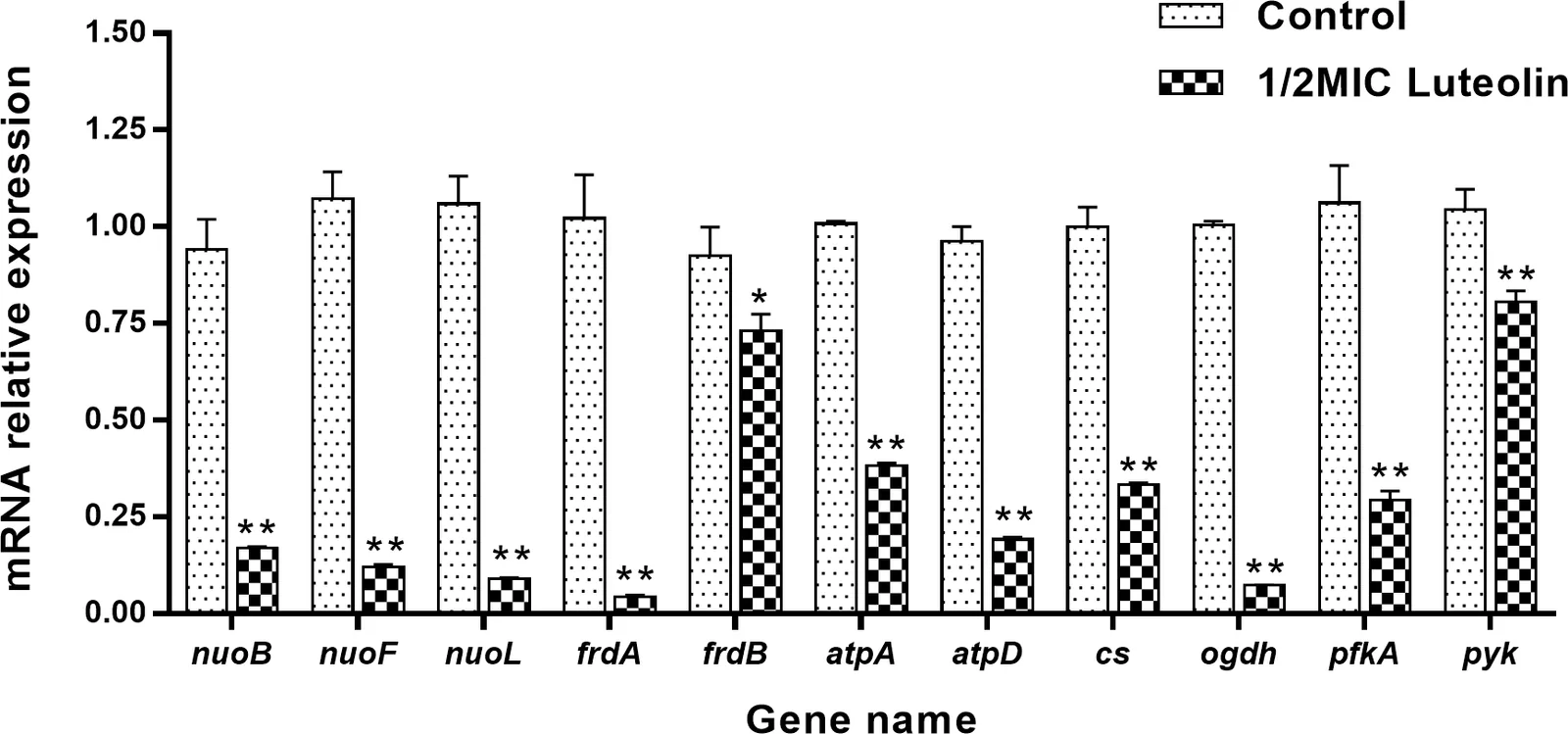
The mRNA relative expression level of genes related to energy metabolism after luteolin treatment (*compared with control, *P < 0.05, **P < 0.01).

To evaluate the correspondence between DEPs and their mRNA expression levels, 11 DEPs related to oxidative phosphorylation, TCA cycle, and glycolysis were selected for transcriptional-level analysis using qRT-PCR. As presented in Fig. 6, the mRNA level of *nuoB*, *nuoF*, *nuoL*, *frdA*, *frdB*, *atpA*, *atpD*, *cs*, *ogdh*, *pfkA*, and *pyk* decreased by 0.17-, 0.12-, 0.09-, 0.04-, 0.74-, 0.38-, 0.19-, 0.33-, 0.07-, 0.29-, and 0.81-folds, respectively. The mRNA levels of these genes all showed a downregulation trend, which was consistent with the results of quantitative proteomics (Table S5). The results provided the genetic evidence showing that luteolin could influence the expression of electron transfer chain complexes, enzymes related to glycolysis, and TCA cycle in *T. pyogenes*.

### Effect of luteolin on the energy metabolism level of *T. pyogenes*

**Fig 7:**
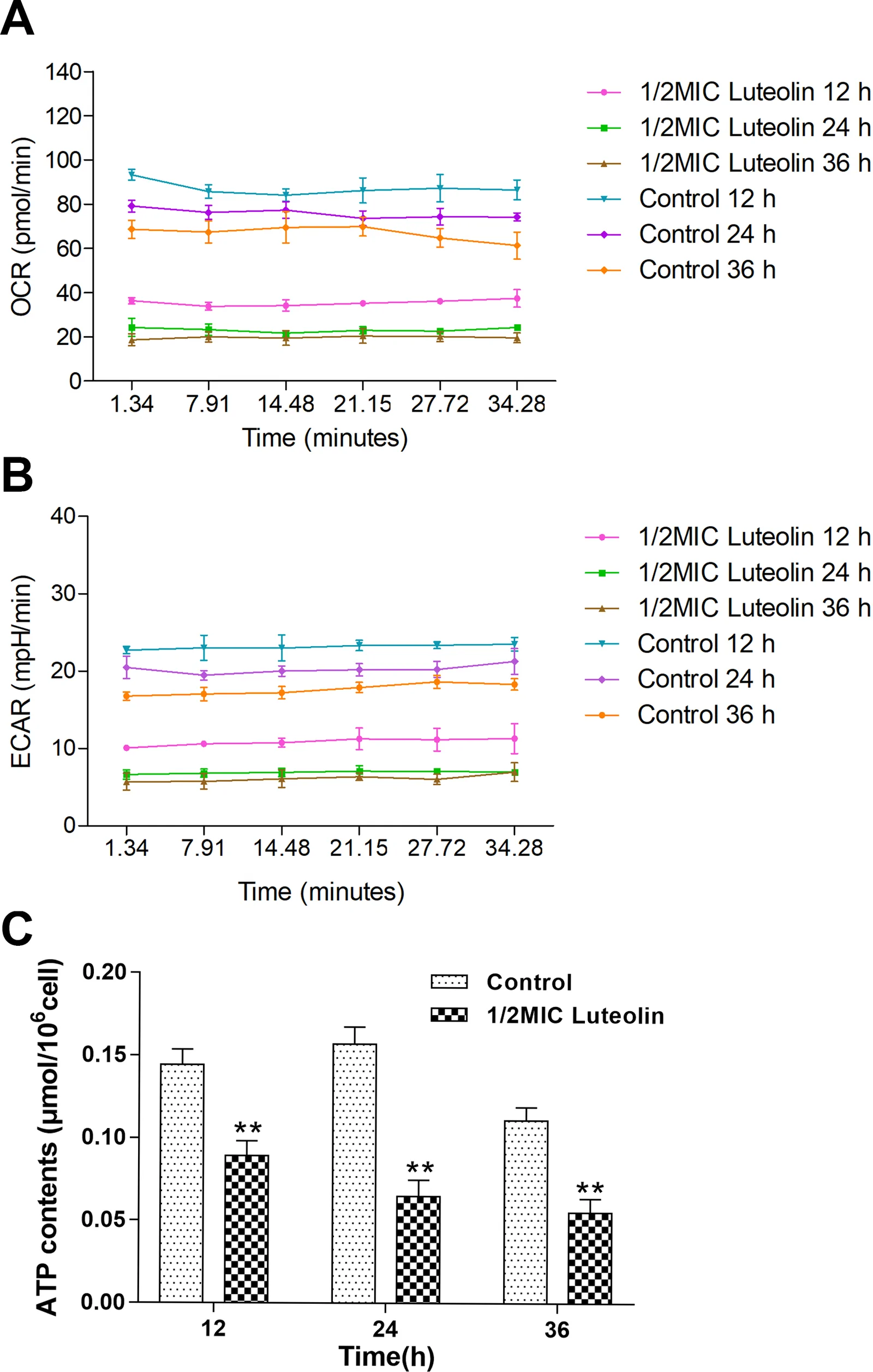
Effects of luteolin on the energy metabolism level of *T. pyogenes*. (**A**) OCR of *T. pyogenes* cells. (**B**) ECAR of *T. pyogenes* cells. (**C**) Intracellular ATP contents of *T. pyogenes* (*compared with control, *P < 0.05, **P < 0.01).

To assess the energy metabolism level changes of *T. pyogenes* induced by luteolin, we measured real-time OCR and ECAR, which were considered as proxies of oxidative phosphorylation and glycolysis. As shown in Fig. 7A and B, luteolin at 78 µg/mL treatment for 12 h significantly impaired the energy metabolism of *T. pyogenes*, reducing the OCR by approximately 70% and the ECAR by 73% compared to the control group. This inhibitory effect persisted throughout the 24 and 36 h treatment periods, indicating substantial suppression of both oxidative phosphorylation and glycolytic activity. Consistent with these findings, intracellular ATP levels decreased markedly by 38.2%, 58.6%, and 50.3% at 12, 24, and 36 h, respectively (Fig. 7C). Collectively, these data provided compelling evidence that luteolin effectively reduces the energy metabolism level of *T. pyogenes*.

### Effect of luteolin on the electron transport chain complex I–V activities

**Fig 8:**
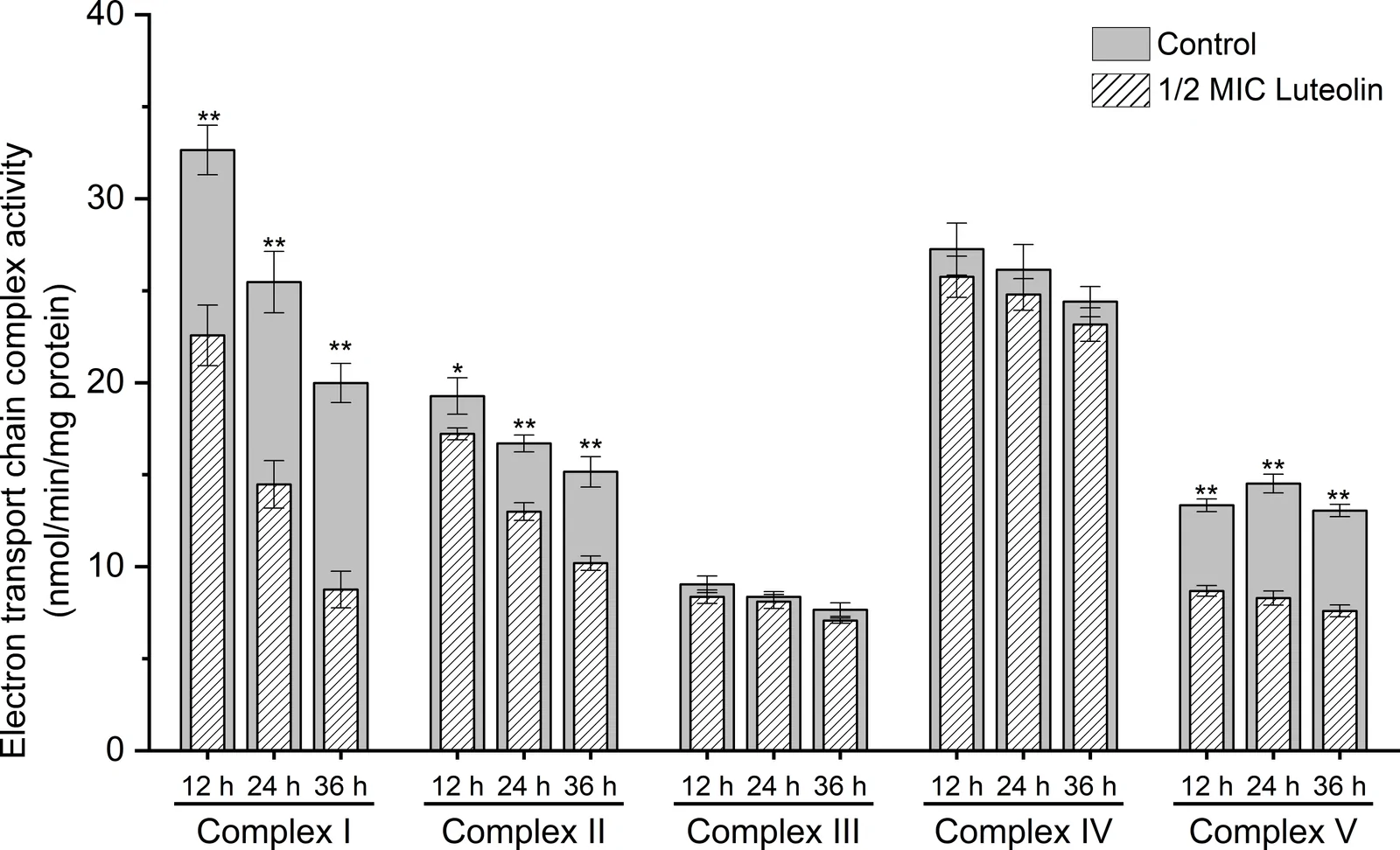
Effects of luteolin on the activity of electron transport chain complexes I–V of *T. pyogenes* (*compared with control, *P < 0.05, **P < 0.01).

The activities of electron transport chain complexes I–V of *T. pyogenes* are shown in Fig. 8. The activity of complex I was decreased by 30.86% after 1/2 MIC luteolin treatment for 12 h (*P* < 0.01), while the activity of complex II was decreased by 10.67% (0.01 < *P* < 0.05) after luteolin treatment for 12 h and further decreased by 22.24% (*P* < 0.01) after 24 h. Notably, luteolin exhibited no inhibitory effect on complex III or IV within the 36 h treatment period. In contrast, luteolin had a great influence on complex V, and its activity decreased by 34.90% after luteolin treatment for 12 h (*P* < 0.01). These results suggested that luteolin primarily targets electron transport chain complexes I, II, and V to disrupt oxidative phosphorylation in *T. pyogenes*.

### Molecular docking and molecular dynamics simulations analysis

**Fig 9:**
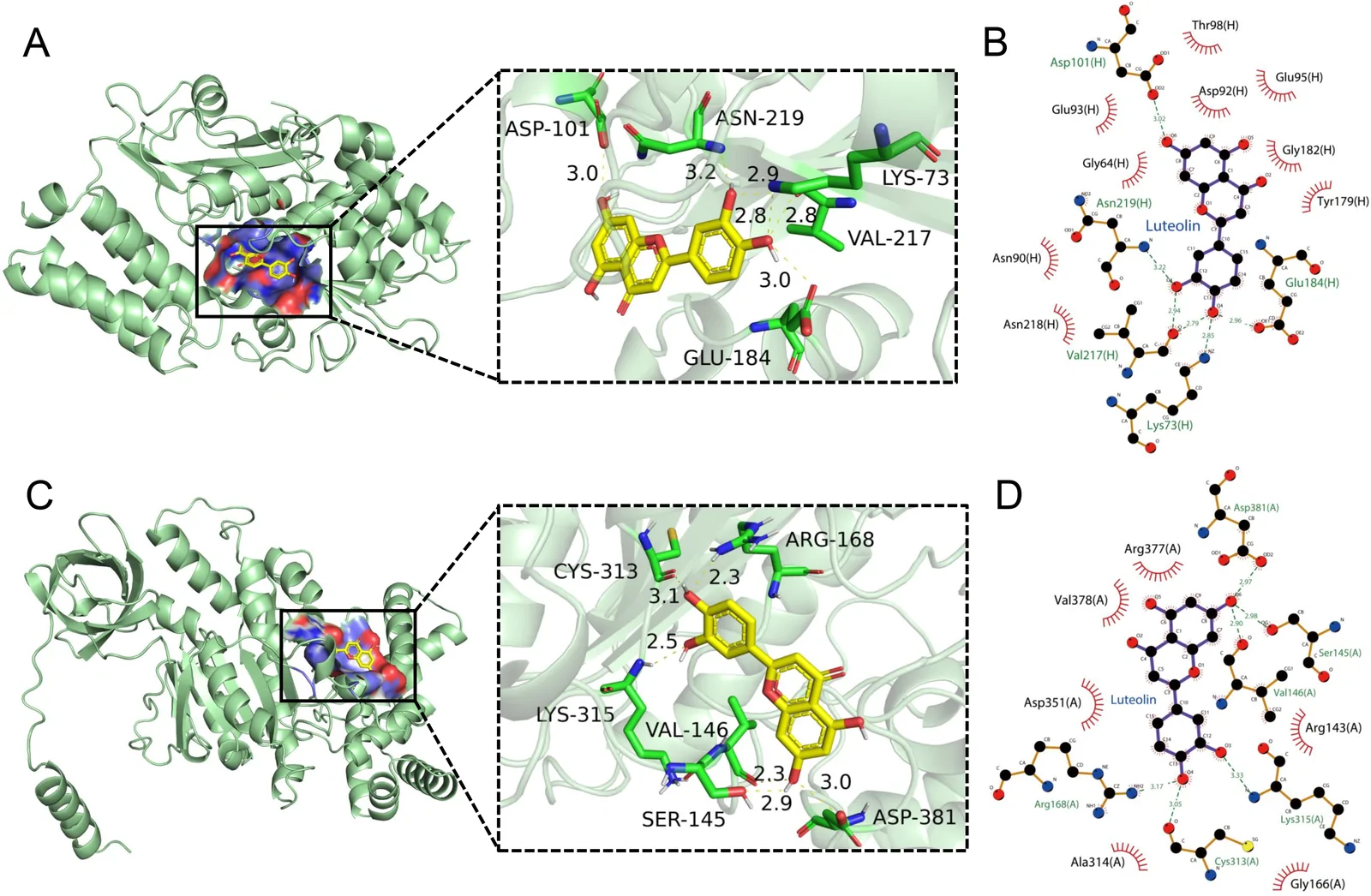
Molecular docking models of luteolin with NuoF and AtpA. (**A**) Three-dimensional (3D) structure model of NuoF and luteolin complex. (**B**) Two-dimensional (2D) schematic diagram of the luteolin binding site on NuoF. (**C**) 3D structure model of AtpA and luteolin complex. (**D**) 2D schematic diagram of the luteolin binding site on AtpA.

Molecular docking was performed to test the potential interaction between luteolin and significantly downregulated electron transport chain complex subunits. Molecular docking calculations were performed in triplicate, and the results of docking scoring are shown in Table S6. It was found that the binding energy of luteolin with 21 electron transport chain complex subunits was all less than −5 kcal/mol. This indicated that luteolin could bind to these subunits stably. As shown in Table S6, luteolin showed lower docking energy with the subunits of electron transport chain complexes I and V compared to those of electron transport chain complex II. Figure 9 displays the interaction pattern of luteolin with NuoF and AtpA, which had the lowest binding energy among the subunits of complexes I and V, respectively. As shown in Fig. 9A and B, luteolin was positioned within the active pocket of NuoF, forming a stable binding interaction. Luteolin established six hydrogen bonds and nine hydrophobic forces with NuoF, yielding a docking score of −8.8 kcal/mol. Similarly, Fig. 9C and D demonstrated that luteolin occupied the active pocket of AtpA, exhibiting strong binding through six hydrogen bonds and six hydrophobic forces, supported by a docking score of −7.2 kcal/mol.

**Fig 10:**
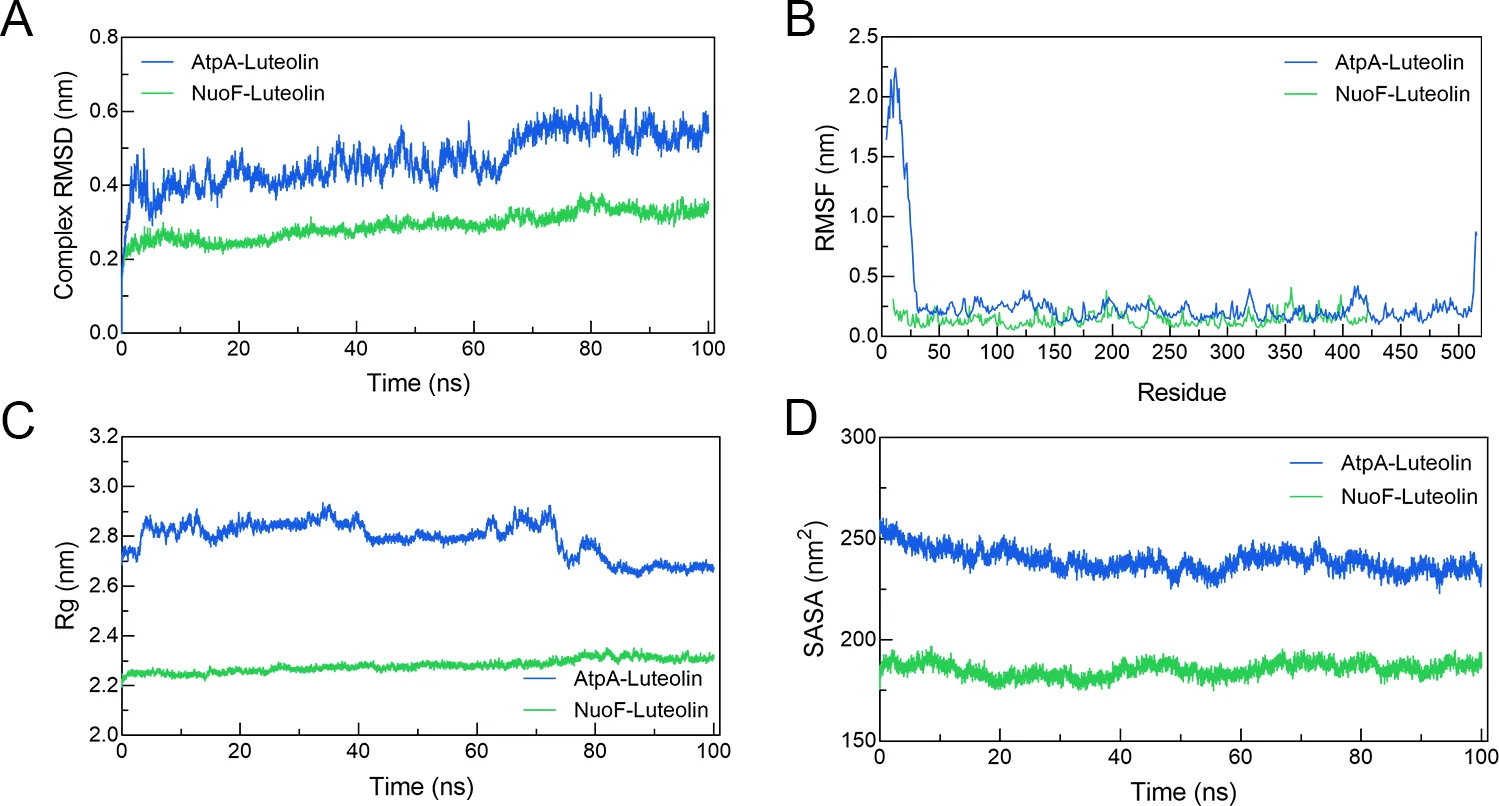
Molecular dynamics analysis of NuoF-luteolin and AtpA-luteolin complexes. (**A**) RMSD, (**B**) RMSF, (**C**) Rg, (**D**) SASA plots for NuoF-luteolin complex (green) and AtpA-luteolin complex (blue).

Additionally, a 100 ns MD simulation was conducted to assess the stability of NuoF-luteolin and AtpA-luteolin complexes. The RMSD analysis revealed both complexes maintained structural stability throughout the simulation period (Fig. 10A). The RMSF profiles indicated greater residue flexibility in AtpA-luteolin, particularly at specific binding sites, while NuoF-luteolin exhibited minimal fluctuations, suggesting more stable binding (Fig. 10B). The Rg analysis demonstrated consistent structural compactness for both complexes, with NuoF-luteolin showing greater stability (Rg value 2.2–2.4 nm) compared to AtpA-luteolin (Rg value 2.6–3.0 nm) (Fig. 10C). The SASA measurements further confirmed structural stability, with minimal fluctuations observed throughout the simulation for both complexes (Fig. 10D). These results collectively demonstrated the relative stability of both NuoF-luteolin and AtpA-luteolin complexes. The NuoF-luteolin complex exhibited superior structural stability, which validated the molecular docking results.

### The affinity of luteolin with NuoF and AtpA

**Fig 11:**
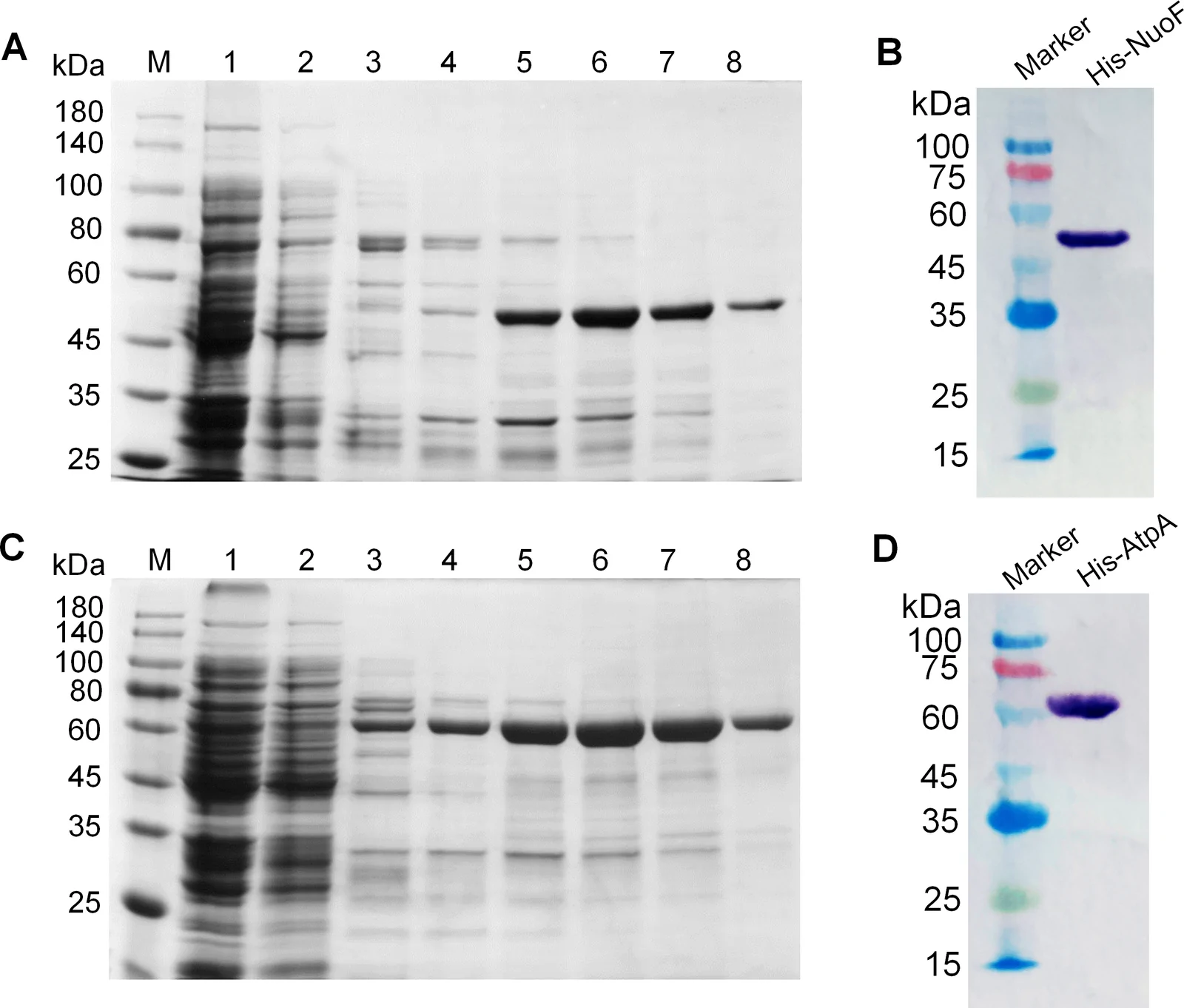
Purification and identification of His-NuoF and His-AtpA fusion proteins. (**A and C**) SDS-PAGE results of His-NuoF (**A**) and His-AtpA (**C**) obtained by immobilized metal affinity chromatography. M, protein marker; 1, flowthrough; 2, washing buffer; 3–8, elution buffer with 80–500 mM imidazole. (**B and D**) Western blot identification results of His-NuoF (**B**) and His-AtpA (**D**).

To investigate the binding affinity between luteolin and the target proteins (NuoF and AtpA), we expressed and purified His-tagged fusion proteins using an *Escherichia coli* expression system. Following induction, the recombinant His-NuoF and His-AtpA proteins were purified by Ni-affinity chromatography, yielding high-purity preparations as confirmed by SDS-PAGE (Fig. 11A and C). Western blot analysis verified the identity of the purified proteins, showing bands at the expected molecular weights (Fig. 11B and D).

**Fig 12:**
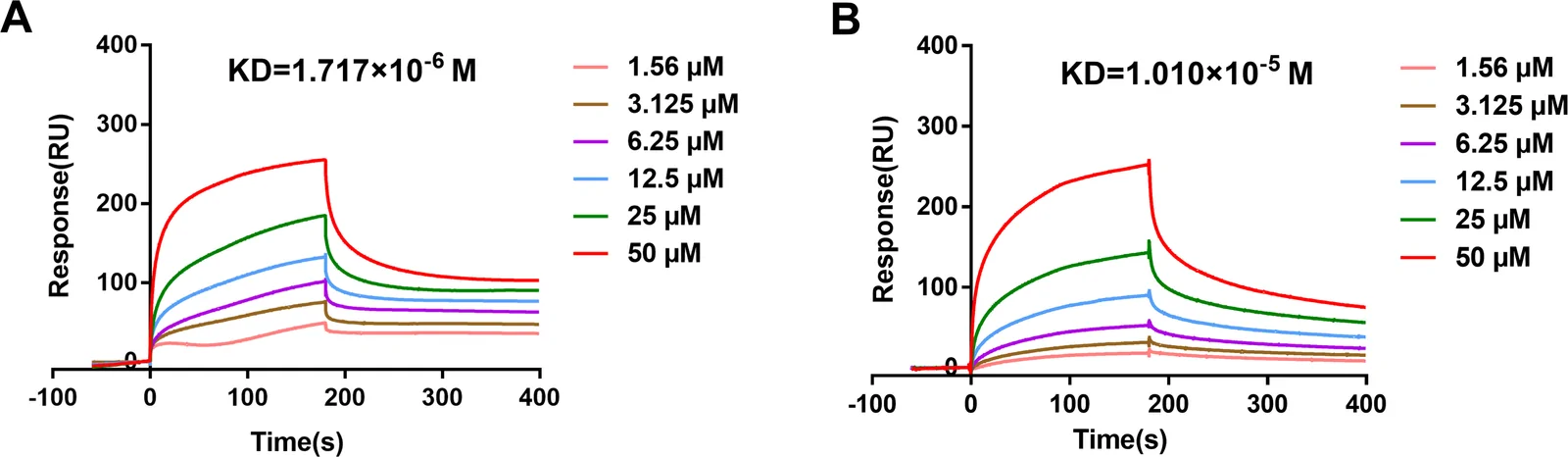
SPR analysis of luteolin binding to His-NuoF and His-AtpA. Luteolin was tested in various concentrations (1.56–50 μM). (**A**) Kinetic fitting curve of luteolin and NuoF. (**B**) Kinetic fitting curve of luteolin and AtpA.

The affinity of luteolin with NuoF and AtpA was detected through SPR technology on the Biacore T200 system. The *K*_*D*_ value revealed strong binding between luteolin and both target proteins, with NuoF exhibiting higher affinity (Fig. 12A; *K*_*D*_ = 1.717 × 10^−6^ M; *k*_*a*_ = 1,291/Ms; *k*_*d*_ = 0.002216/s) compared to AtpA (Fig. 12B; *K*_*D*_ = 1.010 × 10^−5^ M; *k*_*a*_ = 318.4/Ms; *k*_*d*_ = 0.003214/s). These results demonstrated significant binding affinity between luteolin and both proteins, with stronger interaction observed for NuoF compared to AtpA, consistent with molecular docking predictions.

### Effect of luteolin on the enzymatic activities of glycolysis and TCA cycle

**Fig 13:**
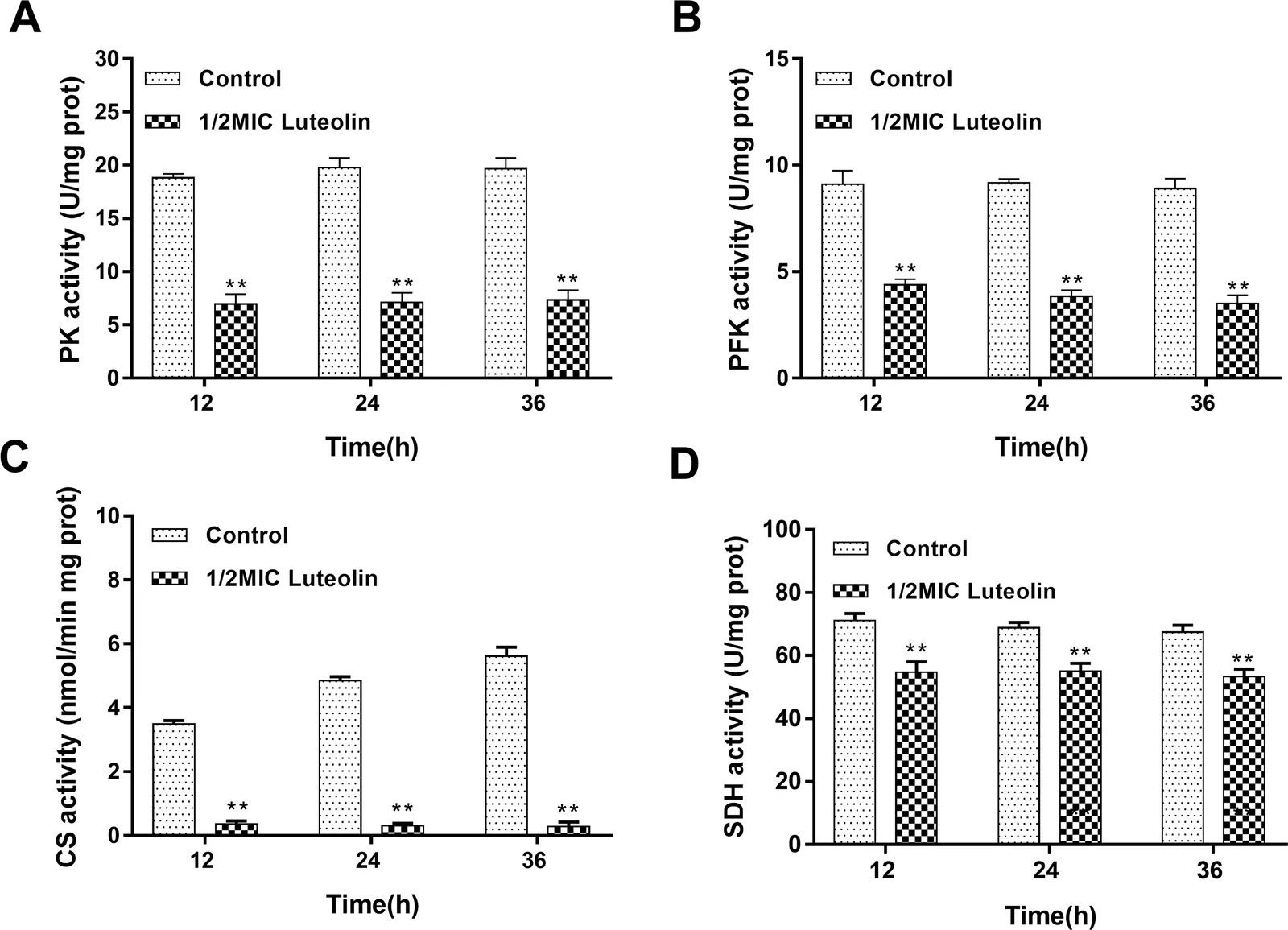
Effects of luteolin on the enzymatic activities involved in glycolysis and TCA cycle of *T. pyogenes*. (**A**) Pyruvate kinase activity. (**B**) Phosphofructokinase activity. (**C**) Citrate synthase activity. (**D**) Succinate dehydrogenase activity (*compared with control, *P < 0.05, **P < 0.01).

KEGG enrichment analysis suggested that luteolin may target glycolysis and the TCA cycle. To validate this, we measured the activities of four key metabolic enzymes, including PK, PFK, CS, and SDH. The results showed that the activities of these enzymes decreased by 62.7%, 60.7%, 95.1%, and 21.1%, respectively after luteolin treatment with *T. pyogenes* for 36 h (Fig. 13). These findings align with the observed downregulation of PK, PFK, CS, and SDH proteins, further supporting the inhibitory effects of luteolin on energy metabolism.

## DISCUSSION

As a significant opportunistic pathogen, *T. pyogenes* can infect a range of animals, including companion, wild, and domestic animals and even humans. Antimicrobial resistance in *T. pyogenes* has become an issue worthy of attention, posing not only substantial challenges to clinical treatment but also potential health risks to occupationally exposed populations (27). Given the rising challenge of antimicrobial resistance worldwide, screening new antibacterial drugs from plant products has emerged as a promising strategy to combat resistant bacteria-mediated infections. Our previous studies found that luteolin exhibited direct antibacterial activity against both susceptible and resistant *T. pyogenes* strains with similar MIC levels (78–156 µg/mL) (23). In this study, we employed high-throughput proteomics and series assays to identify potential novel targets of luteolin and comprehensively elucidate the action mechanism of luteolin.

KEGG enrichment analysis of DEPs can reveal the metabolic pathways significantly affected by drug treatment and suggest the potential action mechanism. The observed upregulation of multiple ribosomal structural proteins in *T. pyogenes* following luteolin treatment may represent a stress adaptation mechanism. This may facilitate enhanced protein synthesis to sustain essential cellular functions under antimicrobial pressure. Although the increased expression of ribosomal structural proteins is beneficial to enhance the ability of bacteria to synthesize protein, it is also a need to be considered that the overexpressed ribosomal proteins will cause assembly errors and eventually lead to the disorder of protein synthesis (28).

Oxidative phosphorylation occurs on the electron transport chain of bacterial cell membranes. It generates the energy currency ATP through coupling with the electron transport chain to supply for various physiological activities of bacteria. The electron transport chain consists of a series of electron carriers, including complexes I–V. Oxidative phosphorylation and electron transport chain are closely coupled. As long as one of them is inhibited, the other one cannot proceed normally. Numerous subunits of electron transport chain complexes were significantly downregulated after luteolin treatment with *T. pyogenes*. This may lead to the inhibition of oxidative phosphorylation and a subsequent decrease in ATP synthesis. The results of OCR, ECAR, and ATP content determination provided compelling evidence that luteolin could effectively reduce the energy metabolism level of *T. pyogenes*. Our previous study demonstrated that luteolin could inhibit the proliferation of *T. pyogenes* and disrupt its morphology. The disruption of cell morphology may be associated with multiple mechanisms, including the direct damaging effect of luteolin on the cell membrane and the downregulation of electron transport chain complexes. Additionally, the inhibition of *T. pyogenes* proliferation by luteolin may also be related to reduced ATP synthesis, as cell division requires energy.

**Fig 14:**
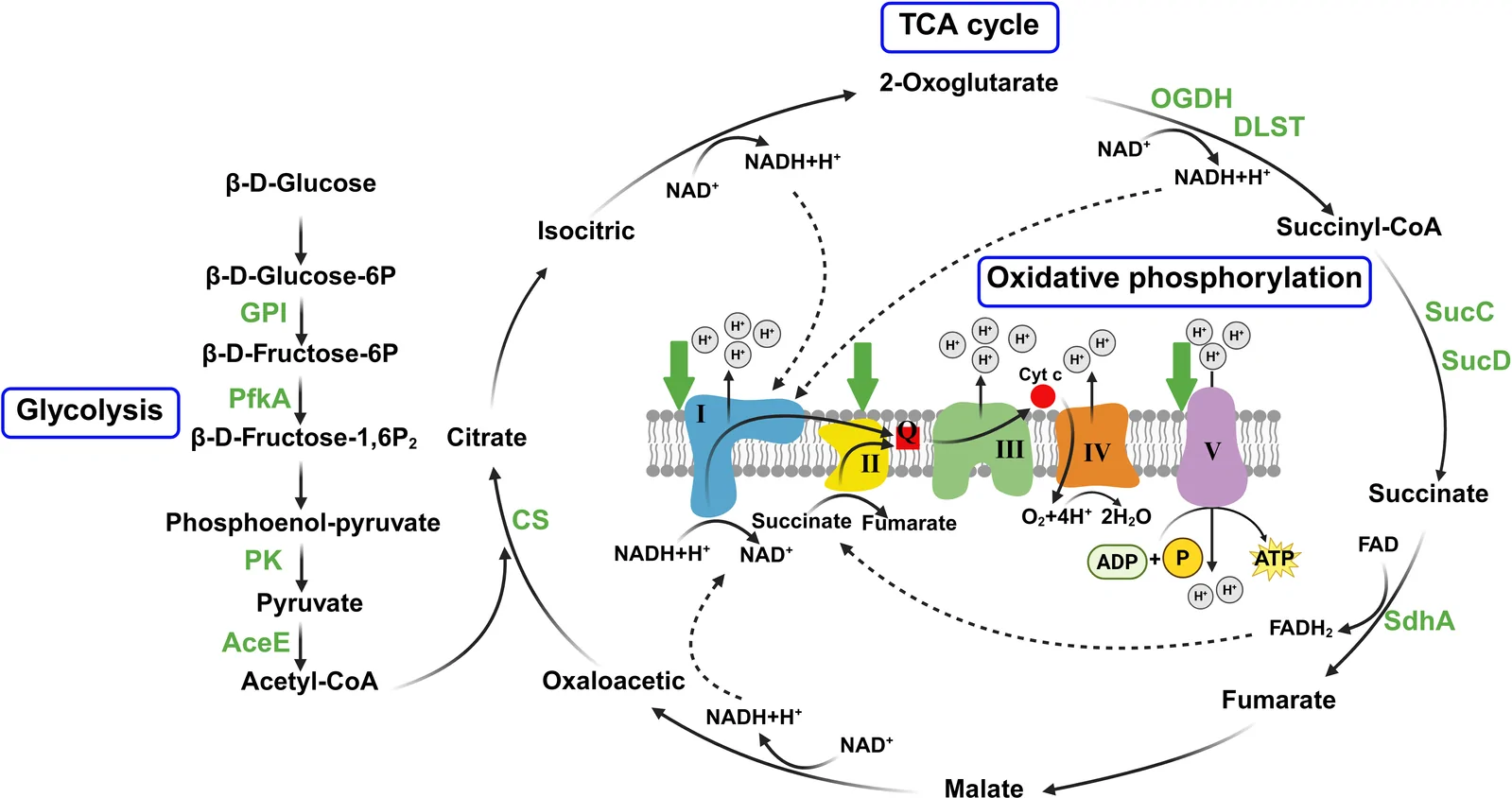
Effect of luteolin on the expression of energy metabolism-related proteins of *T. pyogenes*. Green words and arrows represent significantly downregulated proteins. Luteolin significantly affects energy metabolism-related pathways in *T. pyogenes*. After luteolin treatment on *T. pyogenes*, the expression levels of glucose-6-phosphate isomerase (GPI), 6-phosphofructokinase (PfkA), and pyruvate kinase (PK), as well as pyruvate dehydrogenase subunit (AceE) in the glycolysis pathway were significantly downregulated. In the TCA cycle, 2-oxoglutarate dehydrogenase subunits (OGDH and DLST), succinyl-CoA synthetase subunits (SucC and SucD), and succinate dehydrogenase subunit (SdhA) were significantly downregulated. Furthermore, subunits of electron transport chain complexes I, II, and V in the oxidative phosphorylation pathway exhibited significant downregulation. Created in BioRender. Guo, Y. (2026) https://BioRender.com/cp2seuw.

Natural compounds generally exhibit multitarget characteristics. We also found that multiple enzymes involved in glycolysis and the TCA cycle were significantly suppressed by luteolin. Glycolysis and TCA cycle are crucial metabolic pathways for the oxidative decomposition of carbohydrates. They provide reducing hydrogen for the oxidative phosphorylation that occurs on the electron transport chain. The blockade of oxidative phosphorylation, coupled with the inhibition of the TCA cycle and glycolysis, may suppress ATP synthesis and ultimately lead to bacterial death due to energy depletion (Fig. 14). As flavonoids, sophoraflavanone G and kurarinone could also significantly inhibit the glycolysis and TCA cycle pathways in MRSA, leading to the disruption of the energy metabolism of MRSA and preventing the cells from producing enough energy to survive (29).

Combining the results of proteomics and complexes activity assays, we found that luteolin could suppress electron transport chain complexes I, II, and V. The results of molecular docking showed that luteolin could strongly bind to NuoF and AtpA, which was further confirmed by MD simulation and SPR assay. Accordingly, we hypothesize that the inhibition of the electron transport chain constitutes the primary antibacterial mechanism of luteolin. In addition, the inhibition of multiple enzymes in glycolysis and the TCA cycle may further aggravate energy depletion in *T. pyogenes*. We propose that progressive ATP depletion acts as a key driver of bacterial death. Nevertheless, we cannot fully exclude the possibility that energy metabolic collapse may also be a secondary outcome of severe cellular damage, and further time-series experiments are needed to distinguish whether ATP depletion is the cause or consequence of bacterial death.

Inhibition of energy metabolism has been proposed as a primary antibacterial mechanism for flavonoids (30). The DEPs of *Penicillium italicum* induced by pinocembrin, a flavonoid with a very similar structure to luteolin, were also significantly enriched in the oxidative phosphorylation pathway, and most of them were downregulated. Further studies have shown that pinocembrin could target electron transport chain complexes I, III, IV, and V of *Penicillium italicum* to inhibit oxidative phosphorylation, thus depleting intracellular ATP (31). This suggests that the effects of pinocembrin and luteolin on electron transport chain complexes may be based on the similar chemical structures. Another study showed that two flavonoids, cirsiliol and quercetin, inhibited ATP synthase activity and reduced intracellular ATP contents in MRSA and methicillin-resistant *Staphylococcus epidermidis* (32). Similarly, it was reported that flavonoids such as morin, silymarin, baicalein, silybin, and epicatechin could inhibit ATP synthase of *Escherichia coli*, and morin has the strongest inhibitory effect (33).

In summary, our results indicated that the inhibition of luteolin on the energy metabolism of *T. pyogenes* may be collectively caused by its interference with multiple pathways, including oxidative phosphorylation, glycolysis, and TCA cycle. The inhibitory effect of luteolin on electron transport chain complexes may represent a critical mechanism underlying its antibacterial activity against *T. pyogenes*. Furthermore, our results provided a theoretical foundation for considering electron transport chain complexes as novel targets in the development of drugs against *T. pyogenes* infections.

## Data Availability

The data sets used in the current study are included in this article or supplemental tables.

## SUPPLEMENTAL MATERIAL

Supplemental tables spectrum.01985-26-s0001.docx Tables S1 to S6.

ASM does not own the copyrights to Supplemental Material that may be linked to, or accessed through, an article. The authors have granted ASM a non-exclusive, world-wide license to publish the Supplemental Material files. Please contact the corresponding author directly for reuse.
