# Supplementary material for: Electron transport chain: a crucial target of luteolin in combating Trueperella pyogenes
Source: Microbiol Spectr. 2026 Oct 15;14(11):e01985-26. doi: 10.1128/spectrum.01985-26 (PMC13729196; doi:10.1128/spectrum.01985-26)
Supplement: Supplementary file 1 [file spectrum.01985-26-s0001.docx]

Table S1 Minimum inhibitory concentrations (mg/mL) of different antimicrobial agents against *T. pyogenes* BM-H06-3

| Antimicrobial agents | BM-H06-3 | Susceptibility | ATCC19411 |
| --- | --- | --- | --- |
| Erythromycin | 8 | Resistant | 0.125 |
| Roxithromycin | 16 | - | 0.125 |
| Acetylspiramycin | 32 | - | 2 |
| Tylosin | 32 | - | 0.5 |
| Ciprofloxacin | 16 | Resistant | 4 |
| Enrofloxacin | 8 | - | 4 |
| Streptomycin | 64 | - | 2 |
| Gentamicin | 8 | Intermediate | 2 |
| Amikacin | 8 | - | 1 |
| Thiamphenicol | 4 | - | 2 |
| Florfenicol | 4 | - | 2 |
| Tetracycline | 8 | Intermediate | 1 |
| Chlortetracycline | 1 | - | 0.5 |
| Oxytetracycline | 16 | - | 4 |
| Penicillin | 4 | Resistant | 1 |
| Amoxicillin | 0.25 | - | 0.125 |
| Cephalexin | 2 | - | 2 |
| Luteolin | 156 | - | 78 |

Breakpoints for Erythromycin, Ciprofloxacin, Gentamicin, Tetracycline and Penicillin were recommended by CLSI M45 Coryneform Genera including *Trueperella* species. No breakpoints for the rest of antimicrobial agents are available for this microorganism.

Table S2 Primer sequence and PCR product size

| Primer | Sequence (5’-3’) | Product size (bp) |
| --- | --- | --- |
| *gapA-*F | CGGCGAAGAACGAGGACATCAC | 153 |
| *gapA-*R | GTCGGCAGTGTAGGCGTGAAC |  |
| *nuoB-*F | TGGATATCTACCTGCCTGGATGCC | 118 |
| *nuoB-*R | GCGATCTCCTTGCGATGCTCTC |  |
| *nuoF-*F | ATGTGTATCGCCGTCTGCTTGC | 115 |
| *nuoF-*R | GTCCAGATCGTAATCGCCGTTCG |  |
| *nuoL-*F | GGTCACTGGCTGGCGATTGC | 121 |
| *nuoL-*R | TCCACTCGTACAGCGTCGTCTC |  |
| *frdA-*F | ATGAAGGCCGTGGTGTAGGG | 200 |
| *frdA-*R | TACAGGCCGGGGATCGTAGA |  |
| *frdB-*F | GCAGGTAGAACTCGATGGCG | 200 |
| *frdB-*R | GATCCTCGATGCCCAACAGC |  |
| *atpA-*F | TGACGGGTCTGCCGATCATC | 161 |
| *atpA-*R | CACGCGAGAAACCGAGATGC |  |
| *atpD-*F | ACCATGGCCGAGTACTTCCG | 226 |
| *atpD-*R | GGACGTAGATGGCCTGCAGT |  |
| *ogdh-*F | AAGCATGTCAACCTCGGCATCG | 199 |
| *ogdh-*R | GATTGGTGAGTGTGGCGGTAGTG |  |
| *cs-*F | CGAACAACTGGCGGAGAAGGC | 162 |
| *cs-*R | GAAGACGGGAAGGCTGTGAAGAAG |  |
| *pfkA-*F | GCTTGTTTGTCTAGGCGGTGGAG | 162 |
| *pfkA-*R | ATGGCATCGGTGGCAATCTCAAG |  |
| *pyk-*F | AGGTCCAGGTTCGTGTCGTCTC | 183 |
| *pyk-*R | CGCAGATGTCAGCACCGTAGTTG |  |

Table S3 Amino acid sequences of electron transport chain complex subunits

| Protein name | Amino acid sequence |
| --- | --- |
| NADH-quinone oxidoreductase subunit A (NuoA) | MYSYVPLLVMIIAAGVVAVAGLLVSAIIGPTRHNRVKVANYECGLEATPNAGHEGRFPIKYFLTAMTFIIFDIEVVFLYPWAVSAGYLGVVGVISIAIFVFLITVPFIYEWRRGGLEWE |
| NADH-quinone oxidoreductase subunit B (NuoB) | MGIEEKASGKLLTGIEAVGGWAQARSLWPVTMGLACCAIEMMAFGATRFDASRIGMEVFRASPRHADLMMVSGRVSQKMAPVVRNIYDELADPKWVISMGVCASSGGMFNNYAILQGIDHLVPVDIYLPGCPPRPEMLINAIFELRKHHVEGRPLGEHRKEIARKAEAAALAAIPVEDKKGLLA |
| NADH-quinone oxidoreductase subunit C (NuoC) | MSEKLTDASVVAKRKGMWGVTDTGDTTGFTGNEEISVIAPASQRPYGGWFDEVVDIIIELAQADGFAEQDVIDKVCVDRGQLVIFVKRERLLDVARYLRDDQDLRFEMCLGVSAVHYPMDKGRELHAYIPLFSITHNRMIALEVTCPEDDPHMPSLCSVYPGDDWPEREAFDLMGIIFDGHPGLTRSVMPDDWVGHPQRKDYPLGGIPVEYKGAVIPPPDTRREYN |
| NADH-quinone oxidoreductase subunit D (NuoD) | MKMPHATGAATEESMEEAKIYEAVGGDWGDIAKDAEELGEEHIVVNLGPVHPSTHGVLRVQVELDGERVNEVRAATGFLHTGIEKNMEYRTWTQGVAFCTRMDYVAPIFQEVAYCMAIEKLLGIEDQIPERAKAIRVLLMELTRINSHIVGIGSGGNELGATTMLTLTFRLREDLLRIMEDITGLRMNNEFIRPGGVLEDLPEGGIDYIRELLPGVRNTISEMQDLTMANPIFLKRHVDVAVSPLSALMALSMTGPSIRAAGLPWDLRKTQPYCGYEKYEFDVPVADKCDAYNRIKVKFEECYQSLRIIYQVLDELEQTAGEPVMISDKKIAWPAQLSIAQDGQGTDPAHVREIMTESMESLIHHFKLVTEGFRVPAGQAYATVEHAKGVMGVHLVSDGGTRPYRAHFRDPGFNNLQSLALMAEGGLLADLIIALAAVDPVMGGVDR |
| NADH-quinone oxidoreductase subunit E (NuoE) | MATNYTPEVEARLRADAAEVLARYPQPRSAIMPLLHLIQSEDGFCSPRGITLVADILDLTRAQVSAVATFYSQYRRHPNGEYNVGVCTNALCAVMGGDAIWDELSAYVGVGHDQTTADGKITLEQLECNAGCDYAPVIMVNWEFFDNQTPASAKQIVDDIRAGRDLHPTRGAEKVHTFKEISRVLAGFEDGHVDEGPAAGEASLRGLRIARDKGWSAPKPAGGEETK |
| NADH-quinone oxidoreductase subunit F (NuoF) | MTEFSQPGVLSPVLSDAWDKERSWTLETYRANGGYTAIAKAWQMSEEDGAITNAVKASGLRGRGGAGFPTGLKWSFLPAPDGGPRYLVVNADESEPGTCKDMPFLMANPHLLVEGMMICMLAIGGHHGFIYLRGEVVHVYRRLLAAVREAREAGLLGKGLGPNGDYDLDITVHAGAGAYICGEETALLDSLEGFRGQPRLKPPFPAVEGLYARPTVVNNVESIASIPGIINNGADWFQAMGAATKNSRGHGIFSLSGHVKYPGQYEAPFGITMRELLEMAGGIRDGHELKFYAVGGSSAPLCTPAHLDVPLGYEEVAESGSMLATRAIQVFDETVSVVRVVSRWTDFYQHESCGKCTPCREGTYWMRQLMHRFEAGQAREGEIELLYEIADNIFGRSFCALGDAAATPVRSAIDLFRSEFEAGYTTPAAELFPAEKSVLFREVGVQ |
| NADH-quinone oxidoreductase subunit G (NuoG) | MTTTNATVDLVTIKVDGKEVSVPKGTLIIRAAEKIGVHIPRFCDHPLLAPAAACRQCLVEVASPNRDGVVAKMPKPQPACAVTVSPNMEVYTAASSAVAEKAQHGVMEFLLINHPMDCPVCDKGGECPLQNQAMTDGRATSRFVDVKRTYPKPISVSSQILLDRDRCILCQRCTRFSTQIAGDAFIQLHGRGGGSAGRAVHGLHGSQIGNFDASVLDFGGDAQVVANAFSGPDGQGGREIGFSAGPVETAEMDVTGKPFSSYFSGNVIQICPVGALTSAAYRFRARPFDLVSVPSITEHDASGSAIRVDYRRGTVVRRMAQEDMDVNEAWITDKDRFAFRWQNGPDRLTSPRIAGEEGPISWFEALQTAADGLNAAKAKGVGVITGGRLPLEDAYAYAKFARVALGTNDIDFRTRRASAEEDQFLAACVAGSELGVTYADIERAGHVLTVGLEAEDEIGAVFLRLRKGVLAKSTSVTVISAYGTLGTAKMKARLLPAVPGTEAEVLDAVAAGGAGLFGATFADLSKAGSVILVGERAADTPGALSAAVRLAARTGARLAWIPRRAGDRGAVEAGALGNLLPGGRLVSDAAARIDTAAVWGVDSLPQTPGRSTEQMLAAAKEGQLGALVLAGVELADLPVGAAEALRNVFVVQLEVRHTTTTELADVVLPVAPTAEKGGTFVNWEGRLRPFGQTIVSKQLADRRVLSELAAEMGVNLGLETLKEAVEEMAELRHWDGARGELPNEAPAPAPTVGSGQAVLASWKQMIDAGALQSGEPHLAKTARKPVAVMSAATAEKAGITTRVTITGPGGSLALDVAVVDMPDAVVWVPQNSVGCQIASIGCRAGDVVAISTEVTK |
| NADH-quinone oxidoreductase subunit I (NuoI) | MSEETNAPRFEQPDPALYSPTKKGPVGNFFAPVAGFGVTFSTIFRPTVTEEYPFKKVPTRPRYHGLHKLNRYADGLEKCIGCELCAWACPADAIYVEAAPNSPGAQYSPGERYGRVYQINYLRCIFCAYCIQACPTRALTMSNDYEIAGPTREDMIYEKHQILVPLAKGMLNPPHPMVEGTTDADYYQAAITGPTQAQIDWVKENRPDDPSLATARATSKAEGEN |
| NADH-quinone oxidoreductase subunit J (NuoJ) | MHQLLDISIGETVIFAVVATMMVALAIFGLLIARKAVYSVVSIIFVMVGLAVLYTALEAPFMGVAQVVVYTGAILMMFLFVLMLIGVDSADSTHETLKGQRPVAVLGGIGVAAILIGVVLGATNPDAVGLATANVDGNPVGVARSIFSNHILTLELTGTLLIVAALGAMTLTHRDRIQEPIKQEDRANQAVRDFVAHGHHIGNKPNPGVYAESNSSANPALTAFGEPVEHSVNRVLRVRGQARTLGEVSPATVERITTGGGISGPSTYGQVGRSTLMGMPGEQAPDHDAALARFERSLDPIDAGASPASADEVITAAETTRTHTEKTEEE |
| NADH-quinone oxidoreductase subunit K (NuoK) | MDLMYYVVLAIVLFVVGGATVLTRRNSVIALLGVEIMLNSCNLVLITFSRMWGNLEGQVFAFFVMVVAAAEVVVGLAIIVTIFRTRRTASLDGANLMKH |
| NADH-quinone oxidoreductase subunit L (NuoL) | MIWLTVAIPLASFAILLLAGKAADRWGHWLAIAASTASFLIGAGAAVQLLGLDASERVVETTLYEWIPAVGNFSVDFGTRVDPLSITFVLLVTFVGTLIHVYSVSYMAHDPARRRFFSYLNLFVAAMLLLVLGNSYLVLFFGWEGVGLASYLLISFWNHVPAYATAGKKAFVMNRVGDLGMLIAMMAMVATFESVKFTDVAAGVAQVSEGTASLIGVFLLLAACGKSAQFPLQAWLGDAMAGPTPVSALIHAATMVTAGVYLMVRSGSIYQVAPTATLLVALVGLVTLVFGAVVGAAKDDMKKVLAASTMSQIGYMMLAAGLGPIGAGFAIFHLVTHGFFKANMFLGAGAVMHAMNDEVNIRGFGGLAKHMKITFYTFFAGYLAIIGFPFLSGFFSKDKIIEAAFTGQGALPWIFGIITVLVAGLTAFYMSRLFFAIFLGEERWERHGTNAKHPHDPSPFMWLPMAILALGSVLLGAILNYTGFLTWLEPAIGHAPHADPVLPVPVITGTTLAVVALGVALAWRMYVARPMPKAAPVGNSLVHAARSDLFQDEINEGLFMKPGIALMAGTEALDRSVVDGAVNGIGKATPALGSILSRFQTGYARNYASYVTIGLTLALIVVVASL |
| NADH-quinone oxidoreductase subunit M (NuoM) | MHVQTVSEAGIPWLTSIILLAVIGALVLWLVKPLRQVARPFALGVSLLVGLAYVVALATEFDFASAARVQVVETYSWIPQLGVSLAWGINGMGAVMIALSVFLVPVVILASWNETQGRNSGYLAWVLFLEAIIIGLFAARDLFLFYVLFELMILPIFFMIGRYGKGDKRSTAAMKFLIYSLFGGLIMLIGVIGVYAYGPGGSGGFMIDNLATGSQMSPLAQMWIFVAFFIAFAIKAPMWPVHTWLPDATEEAPAGTSTLLVGILDKMGTFGMIAICLPLFPDAAAQAAPVIIALALVSIIWGAFMAIASDNLMRLIAYTSVSHFGFMVMGIFSGSAIAMAGAIFYMVAHGIGTAALFLAVGFLGERGGSHLISRYGGWQRVTPVLAGAFLISGLATIALPGLSGFIPEYLVLMGTYESVPWAALIGVVGVVMAALYILLPYQRLFTGPRPQVEVGDLDGREKIVMGALIAAMVVLGFFPSLALDAVNPVAEQYSFTISAVIAEGSIK |
| NADH-quinone oxidoreductase subunit N (NuoN) | MNIQIQWMAILPLLIVLGSGVLGTLFEAFVPQRGRRVVQVTLSLTALAAALVVVVWRWTQMQAGGSKITPLSVAVNANGTVAVSLIEDTISLIAQTVIIVGALLSFLVIADRTEIREGAFAAAAATRPGSPEERQYTRLGRQQTEIFPLALFATGGMMAFASAYDLLTLFIALEVLSLPLYVLSATARRKRLLSQEAALKYFILGAFSSAIFLMGAAFIYGSTGTLSLEGTILRLTQGLGIYAPELAAIGTVMLVIGLLFKVGAAPFHAWTPDVYQGAPTPITGFMAAGTKAAAFVALMRVFILLVSPVWPSVNIFMWIIIVATIVIGTVVGLVQTDIKRMLAYSSIAHAGFILIAVNSINIVGNRVSSLIAVPAILFYLLAYAVATVGAFGIVTLVRERDAQGNILGEATKLSHWAGLGKRSPLLAAAMTTFLLSFAGIPLTGGFIGKFEVFKAGIAGNMTILVVLAVVASAATAVFYFRLVQLMFFREPEGENVVVIESEGMSIVSIVLAAIATLALGVVPHFVLEFIMGSFA |
| Fumarate reductase flavoprotein subunit (FrdA) | MSENLIEGLYREGEAIADTKAPAGPLAERWEKRKFAARLVNPANRRKLNVIVVGTGLAGGAGAASLGEMGYNVKAFFYQDSARRAHSIAAQGGINAAKNYKNDNDSTYRLFYDTIKGGDFRARESNVYRLAEVSANIIDQCVAQGVPFAREYGGLLDNRSFGGVQVSRTFYARGQTGQQLLIGAYQALMRQVKVGTVKTYNRHEMVELIVIDGRARGIIARDMATGEIETHLADAVVLATGGYGNVFFLSTNAMGCNTTAIWRAHRKGAYFGNPCYTQIHPTCIPQHGESQSKLTLMSESLRNDGRIWVPKKAEDCKKDPRDIPEEDRDYYLERIYPSFGNLVPRDIASRQAKNMCDEGRGVGPEIGGIARGVYLDFSEAIERMGKDAVSAKYGNLFDMYQQITDENPYEVPMRIYPAVHYTMGGIWVDYDLMSTIPGLYVAGEANFSDHGANRLGASALMQGLADGYFVLPNTINDYLADGPFEKITEDHPAVVEARKSVEERVAFFVNNEGNRSVDSYHKELGHIMWEYCGMERTSAGLKKAIGMIRELRADFWKNVRVPGKAAELNQSLEKAGRLADFLELGELMCIDALHREESCGGHFRAESQTEEGEALRHDDKFAYVAAWEWTGEESAPILHKEDLNYEFVEMKQRSYK |
| Fumarate reductase iron-sulfur subunit (FrdB) | MKINLEFWRQNGSADSGHFDTHTIDNVDETASFLEMLDQLNEELFAEGKEPVAFDSDCREGICGQCGIVINGDPHGPKVTTTCQLHMRTFKDGDTITIEPWRSSAFPVLKDLVVDRSALDRIVQAGGYISVNTGAAPDAHSVPVPKEHADRAFEAAACIGCGACVAACPNGSAMLFTSAKVLHLGLLPQGQPERYDRVVNMLAQHDAEGFGGCTNVGACHEACPKEVPLELISLLNRELMKAQVHGR |
| ATP synthase subunit α (AtpA) | MAELIKPEEIRAALDSFVSSYEPPKAASEEVGHVTLTADGIARVDGLPGAMANELLKFEDDTLGLAMNLEEEEIGVVVLGDFAGIREGMEVRRTGEVLSVPVGDGYLGRVVNPLGEPIDGLGKIAGIDGRRALELQAPGVMMRKSVHEPLQTGLKAIDAMIPIGRGQRQLIIGDRKTGKTALAVDAVLNQKENWESGDPNRQVRCIYVAVGQKGSTIASVRATLEAHGALEYTTIVAAPASDSAGFKYLAPYTGSAIGQHWMYQGKHVLIIFDDLSKQAEAYRSLSLLLRRPPGREAYPGDVFYLHSRLLERCAKLSDELGAGSMTGLPIIETKANDVSAYIPTNVISITDGQIFLQSDLFNSNQRPAVDVGISVSRVGGDAQIKAMKKVAGTLKITLAQYRAQAAFAMFASDLDATTRRQLTRGERLMELLKQPQYTPYAVEDQVASVWAGTHGYLDTIELKDVSKFEAGYLDYLRHNTKVLQTILETGKLEADTEEALAAATQQYLESFTSAYRTHELPTDDVDPAQKSQEQIVRSKRG |
| ATP synthase subunit β (AtpD) | MTDTSLKLANGRIIQVVGAVVDVEFPPDALPEINYALEVEVALPEDETDGTAGTKSMVLEVAQHLGDNIVRTIAMKPTDGLVRGATVRNTGAPISVPVGDITKGHVFNVVGECLNLADGETLEVTERWPIHRTAPNFDQLEGQTKMFETGIKVIDLLTPYVQGGKIGLFGGAGVGKTVLIQEMIQRVAQDHGGVSVFAGVGERTREGNDLIMEMEEAGVLDKTALVFGQMDEPPGVRLRIALSGLTMAEYFRDVQNQDVLLFIDNIFRFTQAGSEVSTLLGRMPSAVGYQPTLADEMGALQERITSTRGHSITSLQAIYVPADDYTDPAPATTFAHLDATTELSRDIASRGLYPAVDPLASTSRILDPQYVGQTHYDVATRVKAILQKNKELQDIISILGVDELSEEDKITVARARRIEQYLSQNTYTAVKFTGVEGSTVPIDETVEAFRRITEGEYDHIPEQAFFNIGGIEDIERAWAKIQAES |
| ATP synthase subunit δ (AtpH) | MRSGSEAALERARDRWAAFVATQPGQEIDLSQQMFSVVDTIKSSTALTSALEGGSRDGDARANLARTLFSGRVADEVTELVESMAREEWSQRNDLQRALEALAVDTILMGARHKGQLSKVEEELYQAMRLYSDHRDLRLAFTTPNITTEARHELAQRLFHRLTPWSVALIRRSVAREHHTIPGLLRRYTDAAARMADHLVAAVTSAIPLTREQEERLVAILSAKYGEPVKVHVSIDTSVIGGLRIYVGDDIIDGTIASRLESVRGAFTN |
| ATP synthase subunit a (AtpB) | MNFSPLTSLPIFLSGGGQGFVPPSVEGEFNPPAFLFQGTPFELNRIVMARMIAAFFLAFILIWYSRHKRLVPNRRLAAVESLLDFSKVKIGEEILGKEHAHRYQPFTMTIFLGLFFMNITGVIPGLQIAGTSLVGMPLIFALFSYFGFIVAGIRAQGGWSFFKGQLFPPGIPWPIYFIMTPIEFLSTFILRPITLTLRLLMNLVAGHLILAICFAGTHYLYFTLSGVVGGITGTLTFLGGIAFMCFEIFVAGLQAYIFAMLTASYISLSISEH |
| ATP synthase subunit b (AtpF) | MISMQELILAEGGFFHILAPGLSELLWGTLAFIIVAVAVYKYAWPAYVETLDERAQKIDEGLREAEQARAEIADSQAKLVDEIRNAQREATGIRENAQDNAKAIIAEAQAKARTEADSLIVGAHRRIDADSEAAMRTLRGDVGVLATELAGRIVGEAIRDEALARRVIDRFLDDLETMTPELKKEAEA |
| ATP synthase subunit c (AtpE) | MIGNIATIGYGLATLGPGIGIGLIGAKNQEATARQPELASFLRVNMFLSMAFAEALGLVGFGAGLVFGA |

Table S4 List of partial differentially expressed proteins

| Accession | Protein name | Fold change | *P* value |
| --- | --- | --- | --- |
| X4R9G3 | 50S ribosomal protein L11 | 1.943 | 1.94753E-05 |
| X4QX64 | 30S ribosomal protein S20 | 1.941 | 2.4819E-06 |
| X4QY52 | 50S ribosomal protein L10 | 1.859 | 1.44443E-07 |
| X4QP06 | 50S ribosomal protein L33 | 1.789 | 8.62934E-05 |
| A0A0M4JXX9 | 30S ribosomal protein S16 | 1.753 | 1.46995E-06 |
| X4QQ52 | 30S ribosomal protein S6 | 1.667 | 1.58308E-05 |
| X4R855 | 30S ribosomal protein S15 | 1.616 | 2.70758E-06 |
| X4RC96 | 50S ribosomal protein L7/L12 | 1.601 | 0.000189913 |
| X4RC71 | 50S ribosomal protein L36 | 1.597 | 0.000665035 |
| X4QMC4 | 30S ribosomal protein S4 | 1.591 | 4.25975E-06 |
| X4QRC5 | 50S ribosomal protein L31 | 1.576 | 0.000748252 |
| X4RC81 | 30S ribosomal protein S8 | 1.569 | 9.44112E-06 |
| A0A2S0RL28 | 50S ribosomal protein L1 | 1.560 | 4.27253E-05 |
| A0A380M8U7 | 50S ribosomal protein L25 | 1.543 | 1.16843E-07 |
| X4QU73 | 50S ribosomal protein L13 | 1.540 | 1.12973E-05 |
| X4QTP7 | 50S ribosomal protein L21 | 1.530 | 2.10993E-05 |
| X4QNX7 | 50S ribosomal protein L5 | 1.510 | 8.65233E-06 |
| X4RC90 | 30S ribosomal protein S10 | 1.492 | 6.02032E-06 |
| X4R882 | 30S ribosomal protein S2 | 1.491 | 5.51684E-06 |
| X4R9E5 | 50S ribosomal protein L22 | 1.461 | 1.57854E-07 |
| A0A2S0RMD9 | 30S ribosomal protein S18 | 1.442 | 1.39834E-06 |
| X4RAM3 | 50S ribosomal protein L9 | 1.435 | 5.68819E-06 |
| X4R9D9 | 50S ribosomal protein L6 | 1.344 | 5.87747E-07 |
| A0A0M4K717 | 30S ribosomal protein S1 | 1.431 | 5.3057E-07 |
| A0A2S0RKT7 | 50S ribosomal protein L17 | 1.430 | 1.06718E-05 |
| X4R9E3 | 50S ribosomal protein L14 | 1.423 | 2.96226E-05 |
| X4RC93 | 30S ribosomal protein S7 | 1.396 | 7.4305E-06 |
| X4QU85 | 30S ribosomal protein S11 | 1.395 | 9.4949E-05 |
| X4R891 | 50S ribosomal protein L19 | 1.387 | 6.32086E-06 |
| X4R0F4 | NADH-quinone oxidoreductase subunit A | 0.817 | 0.026558557 |
| X4QR63 | NADH-quinone oxidoreductase subunit B | 0.423 | 5.77138E-07 |
| X4QWJ7 | NADH-quinone oxidoreductase subunit C | 0.609 | 6.06225E-06 |
| A0A2S0RP31 | NADH-quinone oxidoreductase subunit D | 0.436 | 4.51183E-06 |
| A0A2S0RPN3 | NADH-quinone oxidoreductase subunit E | 0.403 | 4.60191E-05 |
| X4R0G0 | NADH-quinone oxidoreductase subunit F | 0.518 | 4.97098E-08 |
| A0A2S0RQD2 | NADH-quinone oxidoreductase subunit G | 0.462 | 1.73498E-06 |
| A0A2S0RNJ4 | NADH-quinone oxidoreductase subunit I | 0.402 | 3.13142E-06 |
| A0A2S0RNV1 | NADH-quinone oxidoreductase subunit J | 0.743 | 1.34854E-06 |
| X4R0G6 | NADH-quinone oxidoreductase subunit K | 0.770 | 0.001158008 |
| A0A2S0RPE5 | NADH-quinone oxidoreductase subunit L | 0.679 | 0.001094897 |
| A0A2S0RN80 | NADH-quinone oxidoreductase subunit M | 0.811 | 0.000667182 |
| A0A2S0RPT3 | NADH-quinone oxidoreductase subunit N | 0.765 | 0.000361163 |
| X4QU67 | Fumarate reductase flavoprotein subunit | 0.645 | 3.14407E-05 |
| X4QNU7 | Fumarate reductase iron-sulfur subunit | 0.394 | 1.13429E-05 |
| X4QRD5 | ATP synthase subunit beta | 0.723 | 2.78184E-05 |
| A0A3Q9GNA4 | ATP synthase subunit alpha | 0.729 | 4.80796E-06 |
| A0A380MBS0 | ATP synthase subunit delta | 0.820 | 0.000270444 |
| A0A380MD67 | ATP synthase subunit a | 0.784 | 2.11929E-05 |
| A0A380MAD8 | ATP synthase subunit b | 0.761 | 0.000494301 |
| X4QWR4 | ATP synthase subunit c | 0.564 | 0.004373201 |
| A0A3Q9GJZ4 | Citrate synthase | 0.659 | 3.40054E-06 |
| A0A3Q9GP61 | Oxoglutarate decarboxylase | 0.395 | 1.95997E-05 |
| X4QW82 | ATP-dependent 6-phosphofructokinase | 0.739 | 3.14154E-05 |
| X4RAD2 | Pyruvate kinase | 0.400 | 3.84837E-07 |
| A0A2S0RKY9 | Succinate--CoA ligase subunit alpha | 0.473 | 1.57241E-05 |
| A0A380M7X2 | Succinate--CoA ligase subunit beta | 0.458 | 1.29343E-06 |
| A0A2S0RIK6 | Dihydrolipoamide acetyltransferase component of pyruvate dehydrogenase complex | 0.821 | 1.11608E-05 |
| A0A3S9QJ70 | Pyruvate dehydrogenase E1 component | 0.769 | 4.36759E-06 |

Table S5 Transcription level analysis of differentially expressed proteins

| Protein | Gene | TMT fold change | qRT-PCR fold change | Tendency |
| --- | --- | --- | --- | --- |
| NADH-quinone oxidoreductase subunit B | *nuoB* | 0.42↓ | 0.17↓ | consistent |
| NADH-quinone oxidoreductase subunit F | *nuoF* | 0.51↓ | 0.12↓ | consistent |
| NADH-quinone oxidoreductase subunit L | *nuoL* | 0.67↓ | 0.09↓ | consistent |
| Fumarate reductase flavoprotein subunit | *frdA* | 0.64↓ | 0.04↓ | consistent |
| Fumarate reductase iron-sulfur subunit | *frdB* | 0.39↓ | 0.74↓ | consistent |
| ATP synthase subunit alpha | *atpA* | 0.72↓ | 0.38↓ | consistent |
| ATP synthase subunit beta | *atpD* | 0.72↓ | 0.19↓ | consistent |
| Citrate synthase | *cs* | 0.65↓ | 0.33↓ | consistent |
| Oxoglutarate dehydrogenase | *ogdh* | 0.39↓ | 0.07↓ | consistent |
| 6-phosphofructokinase | *pfkA* | 0.73↓ | 0.29↓ | consistent |
| Pyruvate kinase | *pyk* | 0.40↓ | 0.81↓ | consistent |

Table S6 Scoring of molecular docking between luteolin and respiratory chain complex subunit

| Respiratory chain complex of which the subunit belongs | Receptor | Binding energy（kcal/mol） | pKi value (µM) |
| --- | --- | --- | --- |
| Respiratory chain complex I | NADH-quinone oxidoreductase subunit F | -8.8±0.68 | 6.43 |
| Respiratory chain complex I | NADH-quinone oxidoreductase subunit G | -7.3±1.25 | 5.33 |
| Respiratory chain complex V | ATP synthase subunit α | -7.2±0.21 | 5.30 |
| Respiratory chain complex V | ATP synthase subunit a | -7.0±0.85 | 5.13 |
| Respiratory chain complex V | ATP synthase subunit β | -6.9±0.15 | 5.08 |
| Respiratory chain complex V | ATP synthase subunit δ | -6.8±0.23 | 4.96 |
| Respiratory chain complex I | NADH-quinone oxidoreductase subunit L | -6.8±0.31 | 4.96 |
| Respiratory chain complex I | NADH-quinone oxidoreductase subunit N | -6.8±0.49 | 4.96 |
| Respiratory chain complex II | Fumarate reductase flavoprotein subunit | -6.6±0.81 | 4.82 |
| Respiratory chain complex I | NADH-quinone oxidoreductase subunit A | -6.5±0.21 | 4.79 |
| Respiratory chain complex II | Fumarate reductase iron-sulfur subunit | -6.5±0.50 | 4.79 |
| Respiratory chain complex I | NADH-quinone oxidoreductase subunit D | -6.5±0.47 | 4.74 |
| Respiratory chain complex I | NADH-quinone oxidoreductase subunit E | -6.5±0.55 | 4.74 |
| Respiratory chain complex I | NADH-quinone oxidoreductase subunit I | -6.4±0.29 | 4.72 |
| Respiratory chain complex I | NADH-quinone oxidoreductase subunit C | -6.4±0.10 | 4.69 |
| Respiratory chain complex I | NADH-quinone oxidoreductase subunit B | -6.4±0.26 | 4.69 |
| Respiratory chain complex I | NADH-quinone oxidoreductase subunit M | -6.3±0.17 | 4.62 |
| Respiratory chain complex I | NADH-quinone oxidoreductase subunit J | -6.1±0.38 | 4.45 |
| Respiratory chain complex V | ATP synthase subunit c | -5.5±0.53 | 4.03 |
| Respiratory chain complex I | NADH-quinone oxidoreductase subunit K | -5.5±0.12 | 4.01 |
| Respiratory chain complex V | ATP synthase subunit b | -5.4±0.12 | 3.98 |
